# Prediagnosis ultra-processed food consumption and prognosis of patients with colorectal, lung, prostate, or breast cancer: a large prospective multicenter study

**DOI:** 10.3389/fnut.2023.1258242

**Published:** 2023-10-02

**Authors:** Jian-Yuan Pu, Wei Xu, Qian Zhu, Wei-Ping Sun, Jie-Jun Hu, Dong Cai, Jun-Yong Zhang, Jian-Ping Gong, Bin Xiong, Guo-Chao Zhong

**Affiliations:** ^1^Department of Hepatobiliary Surgery, The Second Affiliated Hospital of Chongqing Medical University, Chongqing, China; ^2^Department of Hepatobiliary, Thyroid and Breast Surgery, The People’s Hospital of Liangping District, Chongqing, China; ^3^Department of Epidemiology and Biostatistics, School of Public Health, Chongqing Medical University, Chongqing, China; ^4^Department of Gastrointestinal Surgery, The Second Affiliated Hospital of Chongqing Medical University, Chongqing, China; ^5^Department of Urology, The Second Affiliated Hospital of Chongqing Medical University, Chongqing, China

**Keywords:** ultra-processed foods, prognosis, cancer patients, cohort study, nutritional epidemiology

## Abstract

**Background and aims:**

Whether ultra-processed food consumption is associated with cancer prognosis remains unknown. We aimed to test whether prediagnosis ultra-processed food consumption is positively associated with all-cause and cancer-specific mortality in patients with colorectal, lung, prostate, or breast cancer.

**Methods:**

This study included 1,100 colorectal cancer patients, 1750 lung cancer patients, 4,336 prostate cancer patients, and 2,443 breast cancer patients. Ultra-processed foods were assessed using the NOVA classification before the diagnosis of the first cancer. Multivariable Cox regression was used to calculate hazard ratio (HR) and 95% confidence interval (CI) for all-cause and cancer-specific mortality.

**Results:**

High ultra-processed food consumption before cancer diagnosis was significantly associated with an increased risk of all-cause mortality in lung (HR_quartile 4 vs. 1_: 1.18; 95% CI: 0.98, 1.40; *P*_trend_ = 0.021) and prostate (HR_quartile 4 vs. 1_: 1.18; 95% CI: 1.00, 1.39; *P*_trend_ = 0.017) cancer patients in a nonlinear dose–response manner (all *P*_nonlinearity_ < 0.05), whereas no significant results were found for other associations of interest. Subgroup analyses additionally revealed a significantly positive association with colorectal cancer-specific mortality among colorectal cancer patients in stages I and II but not among those in stages III and IV (*P*_interaction_ = 0.006), and with prostate cancer-specific mortality among prostate cancer patients with body mass index <25 but not among those with body mass index ≥25 (*P*_interaction_ = 0.001).

**Conclusion:**

Our study suggests that reducing ultra-processed food consumption before cancer diagnosis may improve the overall survival of patients with lung or prostate cancer, and the cancer-specific survival of certain subgroups of patients with colorectal or prostate cancer.

## Introduction

Cancer has been become the first or second leading cause of death in the population aged <70 years in most countries, with an estimated 10.0 million cancer deaths in 2020 (1). Currently, up to 43.8 million persons are living with cancer worldwide (2). Thus, it is crucial to determine modifiable risk factors associated with cancer survival.

Ultra-processed foods (UPFs) are described as industrial formulations mostly or totally produced with materials derived from foods and additives, with little or even no whole foods (3). The proportion of calorie generated by UPFs in a person’s total calorie intake each day has reached as high as 25–60%, and their consumption is rapidly increasing globally (4). In addition to their poor nutritional components, UPFs have been adversely related to various health outcomes, including cancer (4). Several large-scale studies have found that increased consumption of UPFs confers increased risks of developing colorectal cancer (5–7), breast cancer (8), head and neck cancers (9), and ovarian cancer (10). Recently, our group also showed a positive relationship of UPF consumption with the risk of developing pancreatic cancer (11). However, to our knowledge, whether UPF consumption is positively related to cancer-related mortality in cancer patients has not been examined.

Colorectal, lung, prostate, and breast cancers are the four most frequently diagnosed cancers worldwide, accounting for 40.4% of new cancer cases and 37.9% of new cancer deaths in 2020 (1). Using the prospective data from the Prostate, Lung, Colorectal, and Ovarian (PLCO) Cancer Screening Trial, we conducted a prospective cohort study to investigate the potential associations of prediagnosis UPF consumption with all-cause and cancer-specific mortality in these four cancer patients.

## Methods

### Study population

Study population of the current study consisted of the participants from the PLCO Cancer Screening Trial, a large multicenter randomized clinical trial that aimed to examine the possible benefits of screening tests or exams in reducing cancer-specific death from these prostate, lung, colorectal, and ovarian cancers. The corresponding study protocol has been published previously (12). In brief, between November 1993 and September 2001, 10 study centers invited American persons between the ages of 55 and 74 to participate in the PLCO Cancer Screening Trial. A total of 154,887 persons were finally included, and they were then randomly divided into the control arm or the screening arm. Participants in the screening arm received the selected screening tests or exams, whereas those in the control arm received the usual care. The PLCO Cancer Screening Trial had been ethically approved by the National Cancer Institute and the Institutional Review Committee of each screening center. Written informed consent was available for each participant.

We took several steps to identify our study population. First, we identified all patients receiving a diagnosis of cancer during the trial (i.e., from trial entry to December 31, 2009) (*n* = 29,225). Then, we excluded the following patients: (1) those who did not to finish a diet history questionnaire (DHQ) (*n* = 7,092); (2) those who had an invalid DHQ (*n* = 1,021) (11); (3) those who did not return a baseline questionnaire (*n* = 363); and (4) those who had been diagnosed with cancer before the DHQ completion (*n* = 5,645). After exclusions, we obtained a cohort of cancer patients completing the DHQ before the diagnosis of the first cancer (*n* = 15,104), which was called hereafter the source population. Finally, we identified patients with colorectal (*n* = 1,100), lung (*n* = 1705), prostate (*n* = 4,336), or breast cancer (*n* = 2,443) from the source population (Supplementary Figure S1). Of note, all included cancer patients were followed up through December 31, 2018; the long follow-up duration allows us to obtain a large number of outcome events of interest and provides an ideal opportunity to determine the potential impacts of UPFs on cancer-related mortality. Importantly, standardized differences of most baseline characteristics between the source and excluded populations were found to be <0.1, reminding us that there was a small possibility of nonparticipation bias, although up to 14,121 participants were excluded (Supplementary Table S1).

### Dietary assessment

Dietary assessment was conducted using the above-mentioned DHQ. The DHQ, a self-administered questionnaire, contains a total of 124 food item and is developed for each participant to collect the frequency and serving size of his or her food consumption during the past year. The performance of this questionnaire had been validated in terms of dietary assessment (13). We approximated daily food consumption by directly multiplying food frequency by serving size, and we estimated daily intakes of nutrients and energy through the DietCalc software (14). Healthy Eating Index-2015, a frequently used diet quality indicator, was calculated for each participant following the method described previously (15). Western diet score was calculated based on the reported method (16) and data availability in the PLCO Cancer Screening Trial, and was defined by eight food groups, namely sugar, animal fat, butter, margarine, eggs, chips, red and processed meat, and salad dressings.

The method used to assess UPF consumption has been mentioned in the literatures (11, 17). Briefly, based on the NOVA classification method (3), two experienced dietitians classified all food and drink items in the DHQ into the four food groups. The specific definition and example for each food group are provided in the literature (3). Our study concentrated on UPFs (group 4), which consist of 64 individual food items (Supplementary Table S2). As per an established categorization method (18), these individual food items were further divided into the following nine food subgroups: ultra-processed fruits and vegetables, cereals, sauces and dressings, meat and meat products, soft drinks, margarine, ultra-processed dairy products, salty snacks, and sugary products.

The overall UPF consumption for a given patient was calculated by summing the amounts consumed of the above-mentioned 64 individual food items. UPF consumption was expressed as servings per day in main analyses primarily based on the USDA Pyramid Servings Database (19), considering different water contents across different food items. Because the consumption of almost all UPFs was initially estimated and recorded in daily grams in the PLCO Cancer Screening Trial, thus UPF consumption was also expressed as grams per day in supplementary analyses for comparison with the results from main analyses. For determining the possible influence of body size, we examined the associations of interest by expressing UPF consumption as daily serving/kilogram body weight. Before the formal data analyses, UPF consumption was adjusted for dietary energy intake with the residual method (20). Notably, given the potential impacts of cancer diagnosis on dietary behaviors, we used UPF consumption before the diagnosis of the first cancer to represent UPF consumption in a patient’s daily life and to perform all data analyses, which exclude the potential that our observed associations are actually caused by reverse causation.

### Ascertainment of mortality outcomes

Mortality status of each cancer patient was ascertained predominantly via an annual study update form. For those who did not return this form, repeat contacts were performed using e-mail or telephone. The ascertainment of mortality status was further supplemented by linkage to the National Death Index. Copies of death certificates were collected for died patients and were used as the primary source of dates and underlying causes of death. For the PLCO cancers, the relevant medical records were additionally reviewed to determine underlying causes of death. The ninth revision of *International Classification of Diseases* was used for coding the causes of death: colorectal cancer (codes 153.0–154.1 and 209.10–209.17), lung cancer (codes 162.2–162.5, 162.8, and 162.9), prostate cancer (codes 185), and breast cancer (codes 174).

### Ascertainment of colorectal, lung, prostate, and breast cancers

Colorectal, lung, prostate, and breast cancers were primarily ascertained through an annual study update form, which was sent to each alive participant for asking if they had received a diagnosis of cancer, and if so, the date and place of diagnosis, and the site and type of cancer. Reports of cancer from the annual study update form were further validated by checking any available medical records. Of note, in the PLCO Cancer Screening Trial, cancer ascertainment also used the data from death certificates and family reports.

### Assessment of covariates

Most baseline characteristics shown in Table 1 were assessed using a self-administered baseline questionnaire. Body mass index (BMI) was calculated by dividing body weight (kg) by height squared (m^2^). Aspirin use referred to regularly taking aspirin or aspirin-containing drugs over the past year. Age at diagnosis and data on treatment, staging, and diagnostic were extracted from patient’s medical records. Notably, data were extracted only for treatments patients received within 1 year of cancer diagnosis. Cancer staging was performed using the AJCC 7th edition staging manual (21). Alcohol consumption was assessed using the DHQ. Physical activity level was expressed as total time of moderate-to-vigorous activity each week, which was estimated based on the information from a self-report supplemental questionnaire.

**Table 1:** Baseline characteristics of study population according to quartiles of energy-adjusted ultra-processed food consumption (daily serving)^a^.

Characteristics	Quartiles of energy-adjusted ultra-processed food consumption (median, servings/day)							
	Colorectal cancer (*n* = 1,100)		Lung cancer (*n* = 1,705)		Prostate cancer (*n* = 4,336)		Breast cancer (*n* = 2,433)	
	Q1 (0.9)	Q4 (7.6)	Q1 (1.0)	Q4 (8.5)	Q1 (1.1)	Q4 (8.5)	Q1 (0.6)	Q4 (6.7)
Age at diagnosis, yrs	73.2 ± 6.3	70.7 ± 6.3	72.8 ± 5.6	71.1 ± 6.1	71.4 ± 5.8	70.1 ± 5.6	70.3 ± 6.3	69.3 ± 6.2
Male, *n* (%)	120 (43.6)	178 (64.7)	205 (48.0)	308 (72.3)	1,084 (100.0)	1,084 (100.0)	0 (0.0)	0 (0.0)
**Racial/ethnic group, n (%)**								
Non-Hispanic White	243 (88.4)	247 (89.8)	387 (90.6)	394 (92.5)	956 (88.2)	996 (91.9)	539 (88.5)	565 (92.9)
Non-Hispanic Black	7 (2.5)	16 (5.8)	16 (3.7)	20 (4.7)	33 (3.0)	55 (5.1)	14 (2.3)	33 (5.4)
Hispanic	0 (0.0)	4 (1.5)	1 (0.2)	4 (0.9)	21 (1.9)	22 (2.0)	10 (1.6)	6 (1.0)
Other race/ethnicity^b^	25 (9.1)	8 (2.9)	23 (5.4)	8 (1.9)	74 (6.8)	11 (1.0)	46 (7.6)	4 (0.7)
Body mass index, kg/m^2^	26.7 ± 4.7	28.7 ± 5.2	25.9 ± 4.5	27.4 ± 4.2	26.5 ± 3.5	28.0 ± 4.1	26.0 ± 5.1	28.6 ± 5.7
Physical activity, min/wk.^c^	100.5 ± 119.7	106.1 ± 123.0	88.0 ± 119.5	88.8 ± 120.5	136.7 ± 127.9	134.4 ± 133.8	117.4 ± 123.5	93.1 ± 114.9
Alcohol intake, g/d	15.7 ± 49.0	11.4 ± 38.2	23.2 ± 60.1	10.0 ± 21.1	21.0 ± 49.5	11.2 ± 24.8	8.1 ± 15.3	4.5 ± 13.4
**Smoking status, n (%)**								
**Current**								
>20 cigarettes/d	7 (2.5)	12 (4.4)	79 (18.5)	115 (27.0)	26 (2.4)	44 (4.1)	9 (1.5)	18 (3.0)
10–20 cigarettes/d	10 (3.6)	17 (6.2)	55 (12.9)	71 (16.7)	38 (3.5)	40 (3.7)	13 (2.1)	24 (3.9)
<10 cigarettes/d	5 (1.8)	5 (1.8)	22 (5.2)	12 (2.8)	15 (1.4)	14 (1.3)	8 (1.3)	21 (3.5)
**Former**								
Stop smoking >15 yrs	71 (25.8)	74 (26.9)	80 (18.7)	61 (14.3)	357 (32.9)	346 (31.9)	139 (22.8)	116 (19.1)
Stop smoking ≤15 yrs	41 (14.9)	65 (23.6)	142 (33.3)	145 (34.0)	158 (14.6)	211 (19.5)	87 (14.3)	113 (18.6)
Never	141 (51.3)	102 (37.1)	49 (11.5)	22 (5.2)	490 (45.2)	429 (39.6)	353 (58.0)	316 (52.0)
Aspirin user, *n* (%)	124 (45.1)	129 (46.9)	201 (47.1)	222 (52.1)	528 (48.7)	574 (53.0)	254 (41.7)	251 (41.3)
History of diabetes, *n* (%)	19 (6.9)	45 (16.4)	15 (3.5)	42 (9.9)	35 (3.2)	79 (7.3)	15 (2.5)	51 (8.4)
History of hypertension, *n* (%)	89 (32.4)	98 (35.6)	129 (30.2)	135 (31.7)	346 (31.9)	359 (33.1)	181 (29.7)	240 (39.5)
Family history of indicated cancer, *n* (%)	32 (11.6)	32 (11.6)	74 (17.4)	90 (21.3)	107 (10.0)	120 (11.1)	110 (18.1)	118 (19.4)
**Trial arm, n (%)**								
Screening	113 (41.1)	106 (38.5)	202 (47.3)	220 (51.6)	561 (51.8)	539 (49.7)	309 (50.7)	300 (49.3)
Control	162 (58.9)	169 (61.5)	225 (52.7)	206 (48.4)	523 (48.2)	545 (50.3)	300 (49.3)	308 (50.7)
Healthy Eating Index-2015	70.0 ± 9.0	59.9 ± 9.4	68.4 ± 9.4	58.2 ± 9.5	70.2 ± 9.0	60.0 ± 9.3	72.9 ± 8.2	63.7 ± 9.1
Energy intake from diet, kcal/d	1390.6 ± 690.2	2277.6 ± 864.7	1466.9 ± 760.1	2362.6 ± 892.6	1597.8 ± 727.2	2558.4 ± 874.7	1291.3 ± 482.2	1838.8 ± 587.4
**Food consumption**								
Vegetable, servings/d^d^	3.4 ± 2.5	4.4 ± 2.8	3.4 ± 2.4	4.3 ± 2.5	3.5 ± 2.3	4.6 ± 2.5	3.7 ± 2.3	4.2 ± 2.3
Fruit, servings/d^d^	2.8 ± 2.3	2.8 ± 2.1	2.4 ± 2.1	2.4 ± 2.7	2.7 ± 2.2	2.8 ± 2.2	3.0 ± 2.1	2.7 ± 1.9
Coffee, servings/d	3.6 ± 3.2	4.1 ± 3.7	5.2 ± 4.1	5.7 ± 4.5	3.9 ± 3.5	4.6 ± 4.0	2.9 ± 2.6	2.9 ± 3.0
Dairy, cups/d	1.2 ± 1.1	1.5 ± 1.1	1.2 ± 1.1	1.6 ± 1.3	1.4 ± 1.3	1.7 ± 1.2	1.2 ± 1.0	1.3 ± 0.9
Fish, g/day	13.5 ± 19.5	16.8 ± 19.6	14.3 ± 19.1	18.2 ± 21.4	14.8 ± 19.3	19.1 ± 25.4	14.5 ± 16.5	15.5 ± 18.0
Whole grain, servings/d	0.9 ± 0.8	1.3 ± 1.0	0.9 ± 0.8	1.3 ± 1.0	1.2 ± 0.9	1.6 ± 1.1	0.9 ± 0.6	1.2 ± 0.8
Red and processed meat, g/d	5.3 ± 5.1	24.3 ± 20.2	7.3 ± 7.9	28.5 ± 26.8	7.1 ± 6.9	29.8 ± 28.4	3.9 ± 3.5	13.6 ± 12.4
**Nutrient intake**								
Dietary fiber, g/d	16.4 ± 9.4	20.8 ± 9.3	15.8 ± 9.3	19.6 ± 8.7	17.4 ± 9.2	22.4 ± 9.7	16.5 ± 8.5	18.5 ± 7.9
Added sugars, tsp./d	7.5 ± 4.1	19.3 ± 13.3	7.5 ± 4.4	20.4 ± 13.4	8.5 ± 4.7	21.5 ± 13.7	7.1 ± 3.8	15.1 ± 9.4
Saturated fatty acids, g/d	13.9 ± 9.0	27.9 ± 13.6	15.0 ± 8.9	30.6 ± 15.8	16.0 ± 9.3	32.4 ± 15.6	13.0 ± 7.3	21.9 ± 10.0
Polyunsaturated fatty acids, g/d	10.2 ± 6.0	18.6 ± 9.1	10.3 ± 5.7	20.3 ± 9.7	11.2 ± 6.0	21.4 ± 9.7	10.1 ± 5.9	16.3 ± 7.3

Q, quartile.

a Values are mean ± standard deviation or counts (percentage) as indicated.

b “Other race/ethnicity” includes Asian, Pacific Islander, or American Indian.

c Total time of moderate-to-vigorous physical activity per week.

d Here, vegetables and fruits not only contained fresh items but also contained processed items and those used in dishes and foods.

### Statistical analysis

Missing data were imputed using the methods described as below. Categorical and continuous covariates with <10% missing data were imputed with the modal value and the median, respectively; the covariate “physical activity level,” which had 32.72% missing data, was imputed with multiple imputation by chained equations (the number of imputations = 25) under the assumption that these data were missing at random (22). Supplementary Tables S3, S4 present the distribution of covariates with missing data before and after imputation in the source population and the included cancer patients, respectively.

We used Cox proportional hazards regression model to calculate hazard ratios (HRs) and their 95% confidence intervals (CIs) of all-cause and cancer-specific mortality in relation to prediagnosis UPF consumption. In this model, follow-up duration was treated as time metric, and was calculated from the date of cancer diagnosis to loss to follow-up, death date, or the end of follow-up, whichever came earlier (Supplementary Figure S2). We examined the proportional hazard assumption using the Schoenfeld residuals (23); as the exposure variable “prediagnosis UPF consumption” was indicated to violate this assumption in analyzing its association with all-cause mortality among breast cancer patients (*P* for global test = 0.020), thus time-dependent Cox regression was used to calculate the corresponding HRs and 95% CIs. To evaluate the potential effects of competing risk bias on the associations of interest, we used competing risk regression to calculate subdistribution HRs and 95% CIs, with other causes of death than death from that cancer studied as competing events. In regression models, prediagnosis UPF consumption was split into quartiles, with the first quartile as the reference group. For examining the linear trends in effect sizes across quantiles, we first assigned the median of each quartile to each patient in that quartile to yield an ordinal variable and then regarded it as a continuous variable in regression models, with its *P* indicating the significance of linear trends. Notably, we chose covariates controlled in multivariable regression models using our causal knowledge of the current literature instead of statistical criteria (24). Specifically, model 1 controlled for age at diagnosis, sex (only for colorectal and lung cancers), race/ethnicity; and model 2 additionally controlled for trial arm, BMI, physical activity, alcohol consumption, smoking status, aspirin use, energy intake from diet, family history of each cancer we studied, history of diabetes, history of hypertension (only for all-cause mortality), and clinical covariates (mainly including cancer stage and treatments, see footnotes of the relevant tables for the exact list of covariates). We used Kaplan–Meier curves to show the cumulative incidence of cancer-related deaths by quarters of prediagnosis UPF consumption. To provide more stable estimates and lower random variability, we classified patients in the first and second quartiles into one group and those in the third and fourth quartiles into another group (25). The difference in cumulative incidence between groups was compared using the log-rank test.

We used restricted cubic spline regression to explore the potential dose–response associations between prediagnosis UPF consumption and all-cause and cause-specific mortality, with the reference level set at 0 servings/day. We used the Akaike’s information criterion and the Bayesian information criterion to determine the number of knots, with the lowest penalized likelihood suggesting the best fitted model. Thus, four knots located at 5th, 35th, 65th, and 95th percentiles were used in exploring dose–response associations with all-cause mortality in prostate cancer patients and with breast-cancer specific mortality in breast cancer patients, whereas three knots located at 10th, 50th, and 90th percentiles were used in exploring other dose–response associations (Supplementary Table S5). A *P*_nonlinearity_ was calculated by testing the null hypothesis that the regression coefficient(s) of the second spline (for three knots) or the second and third splines (for four knots) equal(s) to zero.

We performed a series of sensitivity analyses to determine the stability of our results: (1) excluding patients whose colorectal, lung, prostate, or breast cancer was not the first diagnosed cancer; (2) excluding patients whose colorectal, lung, prostate, or breast cancer was diagnosed ≤2 years after dietary assessment to test the potential influence of the potential reverse causation; (3) excluding patients who died within 30 days or 90 days after cancer diagnosis; (4) excluding patients with extreme UPF consumption (top 2.5% or bottom 2.5%); (5) excluding patients with extreme energy intake (26); (6) repeating the analysis with sex-specific quartiles, since the distribution of UPF consumption was found to be significantly different by sex; (7) additionally adjusting for intakes of fruit, vegetable, coffee, dairy, fish, whole grain, and red and processed meat or intakes of dietary fiber, added sugar, saturated fatty acids, and polyunsaturated fatty acids on model 2; (8) further adjusting for Healthy Eating Index-2015 or Western diet score on model 2 to test whether the observed associations were mediated by diet quality; and (9) additionally adjusted for glycemic index or glycemic load on model 2 to test whether the observed associations were influenced by dietary sugar intake.

We performed several prespecified subgroup analyses to explore whether the observed associations were modified by age at diagnosis (>69 vs. ≤65 years), sex (males vs. females, only in colorectal and lung cancer patients), BMI (≥25 vs. <25), current or formers smokers stopping smoking ≤15 years (yes vs. no), trial arm (screening vs. control arms), and cancer stage (stages I and II vs. stages III and IV). A *P*_interaction_ was obtained via comparing regression models with and without interaction terms prior to the formal subgroup analyses for avoiding the potentially spurious subgroup differences.

To identify the main driver(s) to the observed associations, we examined the associations of each food subgroup consumption with all-cause and cause-specific mortality. Statistical analyses were completed using STATA software version 12.0 (StataCorp). Two-sided *p* < 0.05 was considered statistically significant.

## Results

### Patient characteristics

Regardless of cancer site, in most patients, UPFs contributed to 20–40% of total energy intake before cancer diagnosis (Supplementary Figure S3). Overall, compared with patients in the lowest quartile of prediagnosis UPF consumption, those in the highest quartile had younger age at cancer diagnosis, higher BMI and dietary energy intake while lower alcohol intake and Healthy Eating Index-2015, had a higher possibility of being Non-Hispanic White and present smokers and having histories of hypertension and diabetes; also, patients in the highest vs. the lowest quartiles of prediagnosis UPF consumption had higher intakes of vegetables, dairy, fish, whole grain, red and processed meat, dietary fiber, added sugars, as well as saturated and polyunsaturated fatty acids (Table 1). Supplementary Table S6 presents cancer characteristics and treatment information of included patients.

### Prediagnosis UPF consumption and all-cause and cancer-specific mortality

We observed 643 all-cause deaths and 324 colorectal cancer deaths in colorectal cancer patients during an average follow-up of 8.04 years, 1,525 all-cause deaths and 1,272 lung cancer deaths in lung cancer patients during an average follow-up of 2.95 years, 1,634 all-cause deaths and 254 prostate cancer deaths in prostate cancer patients during an average follow-up of 10.76 years, and 755 all-cause deaths and 189 breast cancer deaths in breast cancer patients during an average follow-up of 10.89 years. After fully adjusting for the potential confounders, high UPF consumption before cancer diagnosis was significantly associated with an elevated risk of all-cause mortality in lung (HR_quartile 4 vs. 1_: 1.18; 95% CI: 0.98, 1.40; *P*_trend_ = 0.021) and prostate (HR_quartile 4 vs. 1_: 1.18; 95% CI: 1.00, 1.39; *P*_trend_ = 0.017) cancer patients (Table 2). When prediagnosis UPF consumption was expressed as daily gram or daily serving/kilogram body weight, the initial results did not change substantially (Supplementary Tables S7, S8). After considering the potential competing risk bias, we obtained similar results for cancer-specific mortality (Supplementary Table S9). In addition, high UPF consumption before cancer diagnosis was found to confer increased risks of lung cancer-specific (HR_quartile 4 vs. 1_: 1.13; 95% CI: 0.93, 1.36) and prostate cancer-specific (HR_quartile 4 vs. 1_: 1.30; 95% CI: 0.84, 2.01) mortality, although the linear trend tests did not reach statistical significance (*P*_trend_ = 0.137 for lung cancer-specific mortality and *P*_trend_ = 0.112 for prostate cancer-specific mortality) (Table 2).

**Table 2:** Hazard ratios (95% confidence interval) for associations of energy-adjusted ultra-processed food consumption (daily serving) before cancer diagnosis with all-cause and cancer-specific mortality in patients with colorectal, lung, prostate, or breast cancer.

Patient group	Quartiles of energy-adjusted ultra-processed food consumption (median, servings/day)				*P*_trend_
**Colorectal cancer patients**					
	Q1 (0.93)	Q2 (2.38)	Q3 (4.21)	Q4 (7.65)	
No. of patients	275	275	275	275	
Person-years	2128.80	2210.20	2215.81	2288.09	
**All-cause mortality**					
No. of deaths	162	170	149	162	
Model 1^a^	1.00 (reference)	1.04 (0.83, 1.29)	0.90 (0.72, 1.13)	1.03 (0.82, 1.30)	0.945
Model 2^b^	1.00 (reference)	1.06 (0.85, 1.33)	0.87 (0.69, 1.10)	0.91 (0.70, 1.18)	0.282
**Colorectal cancer-specific mortality**					
No. of deaths	83	84	77	80	
Model 1^a^	1.00 (reference)	0.95 (0.70, 1.30)	0.86 (0.63, 1.18)	0.93 (0.68, 1.28)	0.653
Model 2^b^	1.00 (reference)	1.10 (0.80, 1.52)	0.92 (0.66, 1.29)	0.96 (0.66, 1.39)	0.621
**Lung cancer patients**					
	Q1 (0.97)	Q2 (2.59)	Q3 (4.55)	Q4 (8.53)	
No. of patients	427	426	426	426	
Person-years	1365.63	1360.01	1159.15	1145.04	
**All-cause mortality**					
No. of deaths	380	372	386	387	
Model 1^a^	1.00 (reference)	0.99 (0.85, 1.14)	1.07 (0.93, 1.23)	1.10 (0.95, 1.27)	0.128
Model 2^b^	1.00 (reference)	0.97 (0.83, 1.12)	1.18 (1.01, 1.38)	1.18 (0.98, 1.40)	0.021
**Lung cancer-specific mortality**					
No. of deaths	316	315	315	326	
Model 1^a^	1.00 (reference)	1.02 (0.87, 1.19)	1.04 (0.89, 1.22)	1.09 (0.93, 1.28)	0.270
Model 2^b^	1.00 (reference)	0.99 (0.84, 1.17)	1.15 (0.97, 1.36)	1.13 (0.93, 1.36)	0.137
**Prostate cancer patients**					
	Q1 (1.14)	Q2 (2.85)	Q3 (4.77)	Q4 (8.52)	
No. of patients	1,084	1,084	1,084	1,084	
Person-years	11943.51	11676.52	11751.54	11303.42	
**All-cause mortality**					
No. of deaths	402	390	425	417	
Model 1^a^	1.00 (reference)	1.01 (0.87, 1.16)	1.14 (0.99, 1.31)	1.28 (1.12, 1.48)	<0.001
Model 2^b^	1.00 (reference)	0.97 (0.84, 1.12)	1.10 (0.95, 1.27)	1.18 (1.00, 1.39)	0.017
**Prostate cancer-specific mortality**					
No. of deaths	58	57	74	65	
Model 1^a^	1.00 (reference)	1.01 (0.70, 1.46)	1.37 (0.97, 1.94)	1.35 (0.94, 1.93)	0.050
Model 2^b^	1.00 (reference)	0.88 (0.60, 1.29)	1.21 (0.84, 1.77)	1.30 (0.84, 2.01)	0.112
**Breast cancer patients**					
	Q1 (0.63)	Q2 (1.98)	Q3 (3.60)	Q4 (6.72)	
No. of patients	609	608	608	608	
Person-years	6712.76	6788.62	6511.92	6489.41	
**All-cause mortality**					
No. of deaths	187	177	189	202	
Model 1^a^	1.00 (reference)	0.90 (0.71, 1.13)	1.02 (0.75, 1.38)	1.17 (0.79, 1.73)	0.163
Model 2^b^	1.00 (reference)	0.85 (0.67, 1.08)	0.89 (0.65, 1.21)	0.85 (0.57, 1.28)	0.697
**Breast cancer-specific mortality**					
No. of deaths	50	39	46	54	
Model 1^a^	1.00 (reference)	0.76 (0.50, 1.16)	0.91 (0.61, 1.36)	1.04 (0.71, 1.54)	0.503
Model 2^b^	1.00 (reference)	0.73 (0.47, 1.12)	0.81 (0.53, 1.24)	0.71 (0.45, 1.10)	0.204

Q, quartile.

a Adjusted for age at diagnosis (years), sex (male, female; only for colorectal and lung cancers), and racial/ethnic group (non-Hispanic White, non-Hispanic Black, Hispanic, others).

b Adjusted for model 1 plus trial arm (screening, control), body mass index (kg/m^2^), physical activity (min/week), alcohol consumption (g/day), smoking status [current (>20 cigarettes/day, 10–20 cigarettes/day, <10 cigarettes/day), former (stop smoking >15 years, stop smoking ≤15 years), never], aspirin use (yes, no), energy intake from diet (kcal/day), family history of indicated cancer (yes, no), history of diabetes (yes, no), history of hypertension (yes, no; only for all-cause mortality), and clinical covariates. For colorectal cancer, clinical covariates were cancer stage (9 categories), surgical resection (yes, no), chemotherapy (yes, no), and radiotherapy (yes, no); for lung cancer, clinical covariates were cancer stage (11 categories), surgical resection (yes, no), chemotherapy (yes, no), and radiotherapy (yes, no); for prostate cancer, clinical covariates were cancer stage (6 categories), Gleason score (2–10 points), PSA level closest to diagnosis (ng/mL), surgical resection (yes, no), radiotherapy (yes, no), cryosurgery or hyperthermia therapy (yes, no), and hormonal therapy (yes, no); for breast cancer, clinical covariates were cancer stage (10 categories), estrogen receptor status (positive, negative, unknown), progesterone receptor status (positive, negative, unknown), HER2 status (0, 1+, 2+, 3+, unknown), and hormone replacement therapy (current use, former use, never use, unknown).

**Figure 1:**
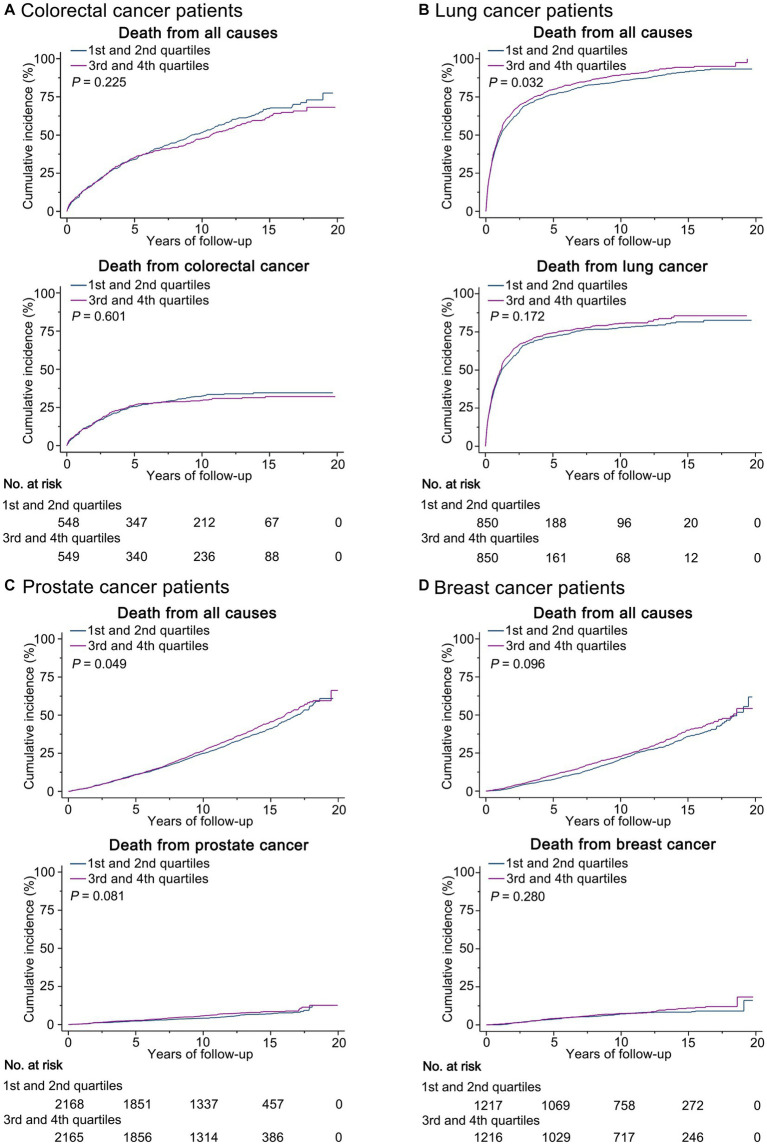
Kaplan-Meier curves show the incidence of deaths from all causes and site-specific cancer by quartiles of prediagnosis ultra-processed food consumption in **(A)** colorectal cancer patients, **(B)** lung cancer patients, **(C)** prostate cancer patients, and **(D)** breast cancer patients.

Kaplan–Meier curves showed that the cumulative incidence of death from all causes was higher among lung (*p* = 0.032) and prostate (*p* = 0.049) cancer patients in the third and fourth quartiles of prediagnosis UPF consumption compared with those in the first and second quartiles, while no significant differences were found for other outcomes of interest (Figure 1).

### Additional analyses

**Figure 2:**
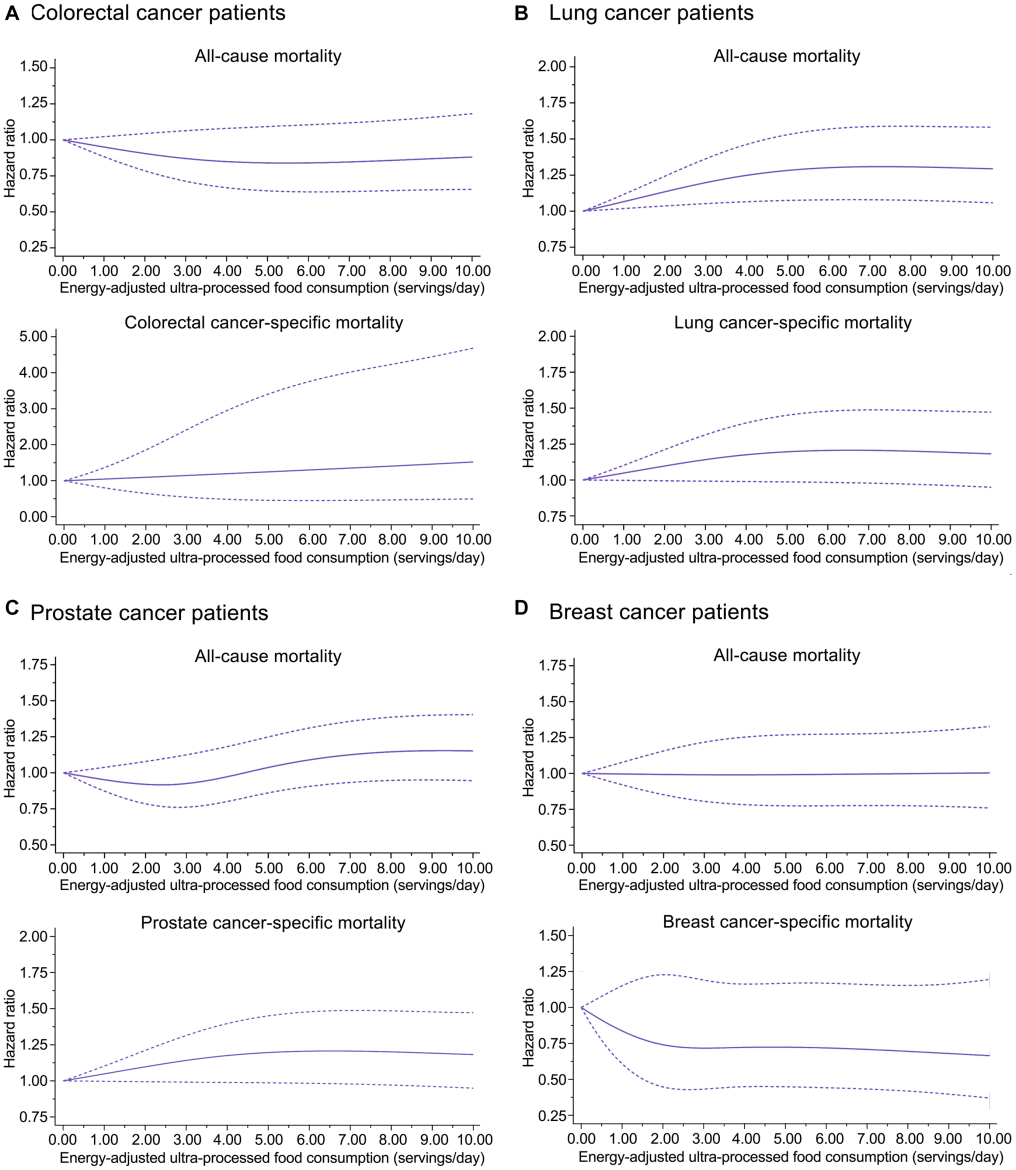
Dose-response analyses on the associations of prediagnosis ultra-processed food consumption with risks of all-cause and cause-specific mortality in **(A)** colorectal cancer patients, **(B)** lung cancer patients, **(C)** prostate cancer patients, and **(D)** breast cancer patients.

A nonlinear dose–response trend was found for the association of prediagnosis UPF consumption with all-cause mortality in lung (*P*_nonlinearity_ = 0.022) and prostate (*P*_nonlinearity_ = 0.042) cancer patients, while such a trend was not found for other associations of interest (Figure 2). The initially observed associations did not change substantially in a wide range of sensitivity analyses (Supplementary Table S10). Interestingly, prediagnosis UPF consumption was positively associated with colorectal cancer-specific mortality among colorectal cancer patients in stages I and II (HR_quartile 4 vs. 1_: 2.52; 95% CI: 1.10, 5.80; *P*_trend_ = 0.033) but not among those in stages III and IV (HR_quartile 4 vs. 1_: 0.96; 95% CI: 0.64, 1.45; *P*_trend_ = 0.674) (*P*_interaction_ = 0.006) (Supplementary Figure S4); moreover, prediagnosis UPF consumption was positively associated with prostate cancer-specific mortality among prostate cancer patients with BMI <25 (HR_quartile 4 vs. 1_: 3.08; 95% CI: 1.25, 7.60; *P*_trend_ = 0.004) but not among those with BMI ≥25 (HR_quartile 4 vs. 1_: 1.16; 95% CI: 0.69, 1.95; *P*_trend_ = 0.577) (*P*_interaction_ = 0.001) (Supplementary Figure S5). No significant interaction was detected in other subgroup analyses (Supplementary Figures S4–S7).

### Prediagnosis consumption of each UPF subgroup and all-cause and cause-specific mortality

Overall, in our study population, main food subgroups contributing to total serving size of UPFs were cereals, soft drinks, meat and meat products, and margarine (Supplementary Figure S8). Higher consumption of sauces and dressings (HR_quartile 4 vs. 1_: 1.17; 95% CI: 1.01, 1.36; *P*_trend_ = 0.046) and ultra-processed fruits and vegetables (HR_quartile 4 vs. 1_: 1.15; 95% CI: 0.99, 1.33; *P*_trend_ = 0.016) before cancer diagnosis conferred a higher risk of all-cause mortality in lung cancer patients, whereas higher consumption of soft drinks (HR_quartile 4 vs. 1_: 1.11; 95% CI: 0.96, 1.28; *P*_trend_ = 0.042), meat and meat products (HR_quartile 4 vs. 1_: 1.16; 95% CI: 1.00, 1.33; *P*_trend_ = 0.019), and ultra-processed fruits and vegetables (HR_quartile 4 vs. 1_: 1.14; 95% CI: 1.00, 1.31; *P*_tren_d = 0.041) conferred a higher risk of all-cause mortality in prostate cancer patients (Supplementary Table S11). Moreover, prediagnosis consumption of sauces and dressings (HR_quartile 4 vs. 1_: 1.23; 95% CI: 1.05, 1.44; *P*_trend_ = 0.033) was positively associated with the risk of lung cancer-specific mortality, and prediagnosis consumption of meat and meat products (HR_quartile 4 vs. 1_: 1.60; 95% CI: 1.08, 2.36; *P*_trend_ = 0.010) and sugary products (HR_quartile 4 vs. 1_: 1.41; 95% CI: 1.00, 2.00; *P*_trend_ = 0.036) was positively associated with the risk of prostate cancer-specific mortality.

In addition, prediagnosis consumption of ultra-processed fruits and vegetables was positively associated with all-cause (HR_quartile 4 vs. 1_: 1.16; 95% CI: 0.91, 1.49) and colorectal cancer-specific mortality (HR_quartile 4 vs. 1_: 1.30; 95% CI: 0.92, 1.85) in colorectal cancer patients, with marginal significance in linear trend tests (both *P*_trend_ = 0.098). Similarly, prediagnosis consumption of meat and meat products (HR_quartile 4 vs. 1_: 1.25; 95% CI: 0.88, 1.79; *P*_trend_ = 0.053) and salty snacks (HR_quartile 4 vs. 1_: 1.31; 95% CI: 0.92, 1.87; *P*_trend_ = 0.064) was positively associated with colorectal cancer-specific mortality. Also, despite the lack of statistical significance in linear trend tests (all *P*_trend_ > 0.05), higher consumption of ultra-processed fruits and vegetables conferred an increased risk of lung cancer-specific mortality (HR_quartile 4 vs. 1_: 1.10; 95% CI: 0.93, 1.29), and higher consumption of meat and meat products conferred increased risks of all-cause (HR_quartile 4 vs. 1_: 1.18; 95% CI: 0.94, 1.49) and breast cancer-specific (HR_quartile 4 vs. 1_: 1.35; 95% CI: 0.87, 2.11) mortality in breast cancer patients.

## Discussion

In this prospective multicenter cohort study, we found that higher consumption of UPFs before cancer diagnosis conferred a higher risk of all-cause mortality in patients with lung or prostate cancer. These findings are mechanistically plausible. First, patients with high UPF consumption are expected to have decreased intake of non-UPFs. Meanwhile, UPFs contain some unfavorable nutritional components, such as added sugars and saturated fatty acids. Indeed, our study observed that patients in the highest quartile of UPF consumption had around one-fold higher intakes of these two nutrients than those in the lowest quartile. Also, a randomized clinical study showed that ultra-processed diets resulted in high calorie intake in the weight-stable inpatients (27). Thus, patients with high UPF consumption may have poor diet quality, which has been demonstrated to be a predictor of poor prognosis in cancer patients (28). Second, UPFs may have some harmful substances generated from packaging materials. For instance, a cross-sectional study found that higher consumption of UPFs led to higher urinary levels of phthalates (29), a class of chemicals frequently applied in food packaging. Importantly, experimental studies have found that phthalates promote the proliferation of prostate cancer cells by activating MAPK/AP-1 pathway (30), and that di(2-ethylhexyl) phthalate weakens the ability of camptothecin, a cancer chemotherapy agent, to inhibit lung cancer cell growth via reducing DNA damage and activating Akt/NF-κB pathway (31). Third, UPFs possibly have some neo-formed chemical substances generated during biological, chemical, and/or physical industrial processes UPFs experience (e.g., acrylamide) (32). Of note, an early prospective study had observed that prediagnosis acrylamide exposure was inversely associated with the overall survival in women with postmenopausal breast cancer (33). In fact, the International Agency for Research on Cancer has classified acrylamide as a Group 2A carcinogen. Fourth, food additives may be added to UPFs to increase their palatability and shelf life. However, mounting evidence has shown their adverse effects on cancer survival. For instance, a recent pooled analysis revealed a positive association of the exposure to titanium dioxide, a whitening and brightening food additive, with the risk of lung cancer-specific mortality (34). Finally, UPFs have been suggested to have higher glycemic index than other NOVA-defined food groups (i.e., groups 1–3) (35). Meanwhile, extensive studies have shown that dietary glycemic index is positively associated with risks of developing colorectal, lung, prostate, and breast cancers (36–39). Moreover, a prospective cohort study of 8,932 breast cancer patients showed that higher dietary glycemic index after breast cancer diagnosis conferred poorer overall survival (40). Thus, glycemic index may mediate the observed association between UPF consumption and all-cause mortality in patients with lung or prostate cancer. However, this explanation seems to be not supported by the observation that our initial results remained after further adjustment for glycemic index.

Our subgroup analysis observed that high UPF consumption before cancer diagnosis conferred an increased risk of death from colorectal cancer among patients in the early stage of colorectal cancer but not among those in the advanced stage. The specific mechanisms behind this phenomenon are unknown. A possible explanation is that the possibly adverse impacts of UPFs on colorectal cancer-specific mortality may have been masked or severely diluted by the impacts of poor condition of cancer patients in the advanced state, given that advanced stage is a strong risk factor for cancer survival. A similar explanation could be applied to the observation that prediagnosis UPF consumption was positively related to prostate cancer-specific mortality in patients with BMI <25 but not in those with BMI ≥25, because excess body weight and/or its related unhealthy lifestyle (41) are also strong predictors of poor prognosis of prostate cancer patients (42, 43). Nevertheless, we cannot exclude the possibility that the observed interactions between prediagnosis UPF consumption and cancer stage and BMI are chance findings. Hence, the results from our subgroup analyses should be treated with caution, and need to be further confirmed.

Several limitations should be acknowledged. First, dietary assessment was performed only at a single time point before cancer diagnosis, resulting in that our findings might be influenced by nondifferential bias, because a person’s dietary habits possibly change over time. Nevertheless, it has been demonstrated that the approaches only using the most recent diet or the baseline diet generally yield a weaker association than do these using the cumulative averages (44). Second, though we had controlled some potential confounders, our findings might still be susceptible to residual cofounding owing to undetected or unrecognized confounders. For example, we failed to control cancer stage and treatments and the lifestyles after diagnosis in multivariable analyses for breast cancer, as these clinical data were not collected for this cancer. Moreover, our results cannot establish a causal association between prediagnosis UPF consumption and cancer-related mortality, given the observational nature of our study. Third, death certificates were used as the primary source for determining underlying causes of death. Notably, causes of from death certificates could be misclassified in some conditions (45), thus our results on cancer-specific mortality might be affected by misclassification bias. Finally, the average age at diagnosis of included patients was about 70 years; about 90% of them were Non-Hispanic White; and about half of them were aspirin users or current or former smokers. These factors decrease the generalizability of our results to some extent. Hence, our findings are likely not generalize to other populations.

In summary, high UPF consumption before cancer diagnosis is associated with an elevated risk of all-cause mortality in patients with lung or prostate cancer and cancer-specific mortality of certain subgroups of patients with colorectal or prostate cancer, indicating that reducing UPF consumption before cancer diagnosis may improve the overall or cancer-specific survival of these cancer patients. More studies are warranted to validate our findings in other populations and settings.

## Data availability statement

The raw data supporting the conclusions of this article will be made available by the authors, without undue reservation.

## Ethics statement

The studies involving humans were approved by the US National Cancer Institute and the Institutional Review Committee of each screening center. The studies were conducted in accordance with the local legislation and institutional requirements. The participants provided their written informed consent to participate in this study.

## Author contributions

J-YP: Conceptualization, Writing – original draft, Writing – review & editing. WX: Methodology, Software, Validation, Writing – review & editing. QZ: Formal analysis, Methodology, Software, Writing – review & editing. W-PS: Software, Validation, Visualization, Writing – review & editing. J-JH: Methodology, Writing – review & editing. DC: Investigation, Methodology, Writing – review & editing. J-YZ: Investigation, Writing – review & editing. J-PG: Investigation, Writing – review & editing. BX: Conceptualization, Writing – review & editing, Formal analysis, Investigation, Methodology, Software. G-CZ: Conceptualization, Project administration, Supervision, Writing – review & editing.
