# Supplementary material for: Prediagnosis ultra-processed food consumption and prognosis of patients with colorectal, lung, prostate, or breast cancer: a large prospective multicenter study
Source: Front Nutr. 2023 Oct 2;10:1258242. doi: 10.3389/fnut.2023.1258242 (PMC10577176; doi:10.3389/fnut.2023.1258242)
Supplement: Supplementary file 1 [file Data_Sheet_1.doc]

***Supplemental Materials***

**Prediagnosis ultra-processed food consumption and prognosis of patients with colorectal, lung, prostate, or breast cancer: a large prospective multicenter study**

Jian-Yuan Pu, et al.

**Table of contents**

Figure S1. The flow chart of identifying eligible cancer patients ……………………………………… 3

Figure S2. The timeline and follow-up scheme of our study …………………………………………... 4

Figure S3. The distribution of proportion (%) of energy brought by ultra-processed foods in daily energy intake …………………………………………………………………………………………….5

Figure S4. Subgroup analyses on the associations of energy-adjusted ultra-processed food consumption before cancer diagnosis with all-cause and colorectal cancer-specific mortality in colorectal cancer patients …………………………………………………………………………………………………. 6

Figure S5. Subgroup analyses on the associations of energy-adjusted ultra-processed food consumption before cancer diagnosis with all-cause and prostate cancer-specific mortality in prostate cancer patients ………………………………..................................................................................................... 7

Figure S6. Subgroup analyses on the associations of energy-adjusted ultra-processed food consumption before cancer diagnosis with all-cause and lung cancer-specific mortality in lung cancer patients …………………………………………………………………………………………………. 8

Figure S7. Subgroup analyses on the associations of energy-adjusted ultra-processed food consumption before cancer diagnosis with all-cause and breast cancer-specific mortality in breast cancer patients …………………………………………………………………………………………………. 9

Figure S8. Proportion (%) of each food subgroup in total energy-unadjusted serving size of ultra-processed foods in four cohorts of cancer patients ……………………………………………… 10

Table S1. Comparison of baseline characteristics between the source and excluded populations ……………………………………………………………………………………………. 11

Table S2. Ultra-processed foods in each food subgroup with assigned serving sizes and energy values …………………………………………………………………................................................. 12

Table S3. Distribution of covariates with missing data before and after imputation in the source population …………………………………………………………………………………………….. 14

Table S4. Distribution of clinical covariates with missing data before and after imputation in cancer patients ………………………………………………………………………………………………... 15

Table S5. The Akaike’s information criterion and Bayesian information criterion values for all associations investigated when the number of knots was set at 3, 4, and 5 ………………………………………………………………………………………………………. 18

Table S6. Cancer characteristics and treatment information of included patients according to quartiles of energy-adjusted ultra-processed food consumption (daily serving) before cancer diagnosis ……………………………………………………………………………………………… 19

Table S7. Hazard ratios (95% confidence interval) for associations of energy-adjusted ultra-processed food consumption (daily gram) before cancer diagnosis with all-cause and cancer-specific mortality in patients with colorectal, lung, prostate, or breast cancer ………………………………………………………………………………. …………………22

Table S8. Hazard ratios (95% confidence interval) for associations of energy-adjusted ultra-processed food consumption (daily serving/kilogram body weight) before cancer diagnosis with all-cause and cancer-specific mortality in patients with colorectal, lung, prostate, or breast cancer …………………………………………………………………………………………………. 24

Table S9. Subdistribution hazard ratios (95% confidence interval) for the association of energy-adjusted ultra-processed food consumption (daily serving) before cancer diagnosis with cancer-specific mortality in patients with colorectal, lung, prostate, or breast cancer ………………………………………………………………………......................................... 26

Table S10. Sensitivity analyses on the associations of energy-adjusted ultra-processed food consumption (daily serving) before cancer diagnosis with all-cause and cancer-specific mortality in patients with colorectal, lung, prostate, or breast cancer …………………………………………………………………………………………………. 28

Table S11. Explanatory analyses on the associations of energy-adjusted consumption (daily serving) of individual ultra-processed food groups before cancer diagnosis with all-cause and cancer-specific mortality in patients with colorectal, lung, prostate, or breast cancer ………………………………………………………………………......................................... 32


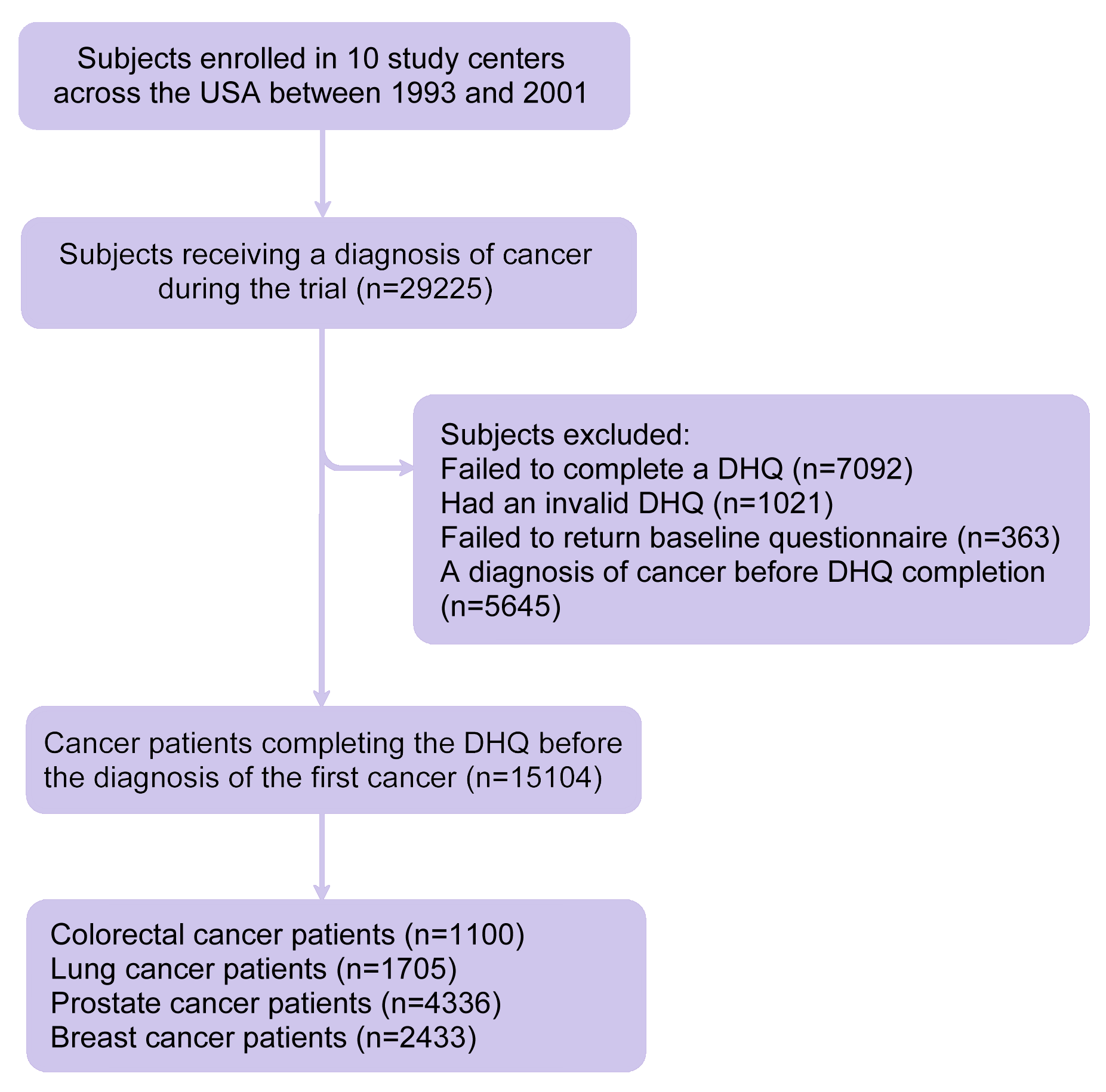


**Figure S1.** The flow chart of identifying cancer patients eligible for our study. DHQ, diet history questionnaire.


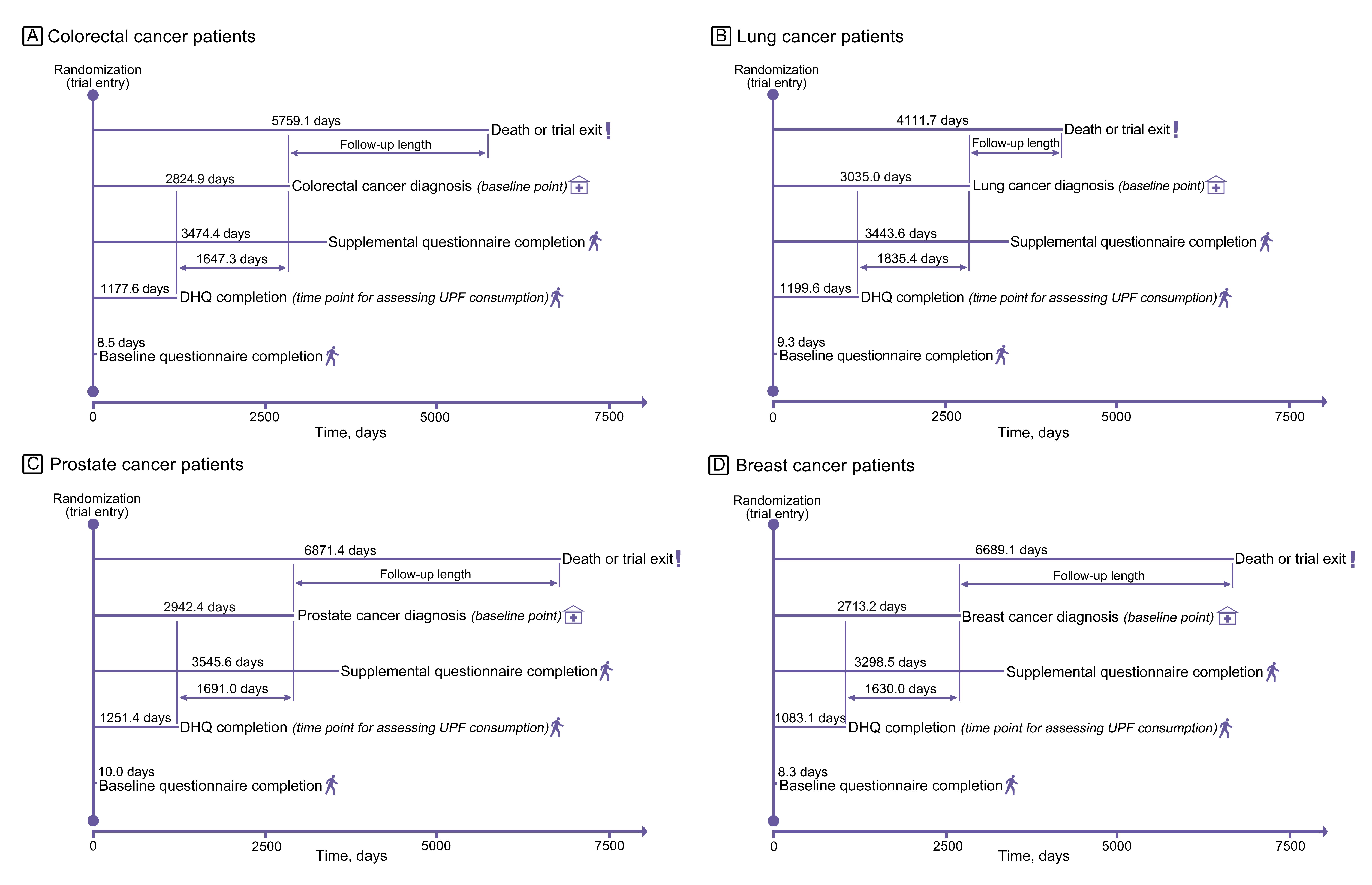


**Figure S2.** The timeline and follow-up scheme of our study. The baseline point was set at the date of cancer diagnosis.


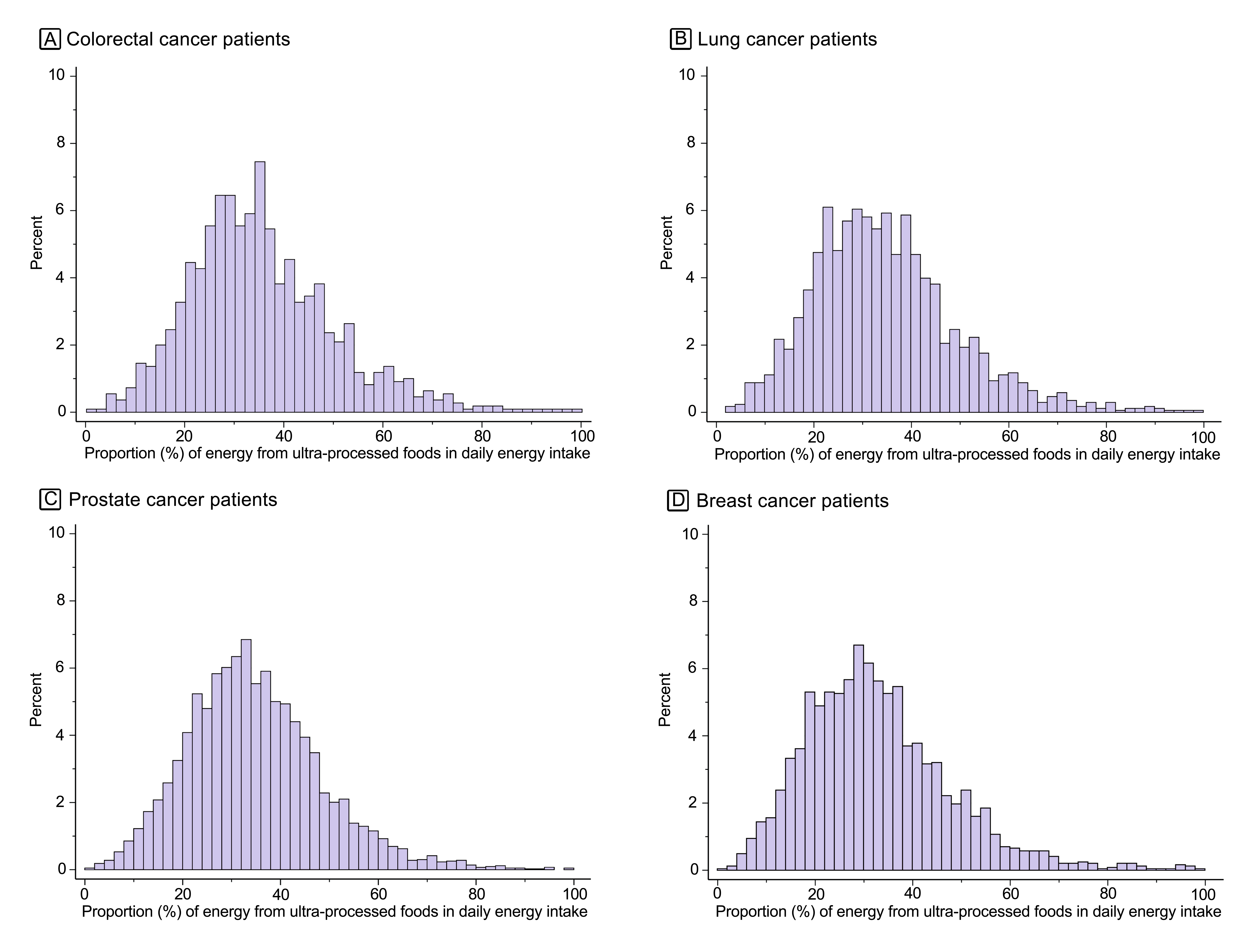


**Figure S3.** The distribution of proportion (%) of energy brought by ultra-processed foods in daily energy intake.


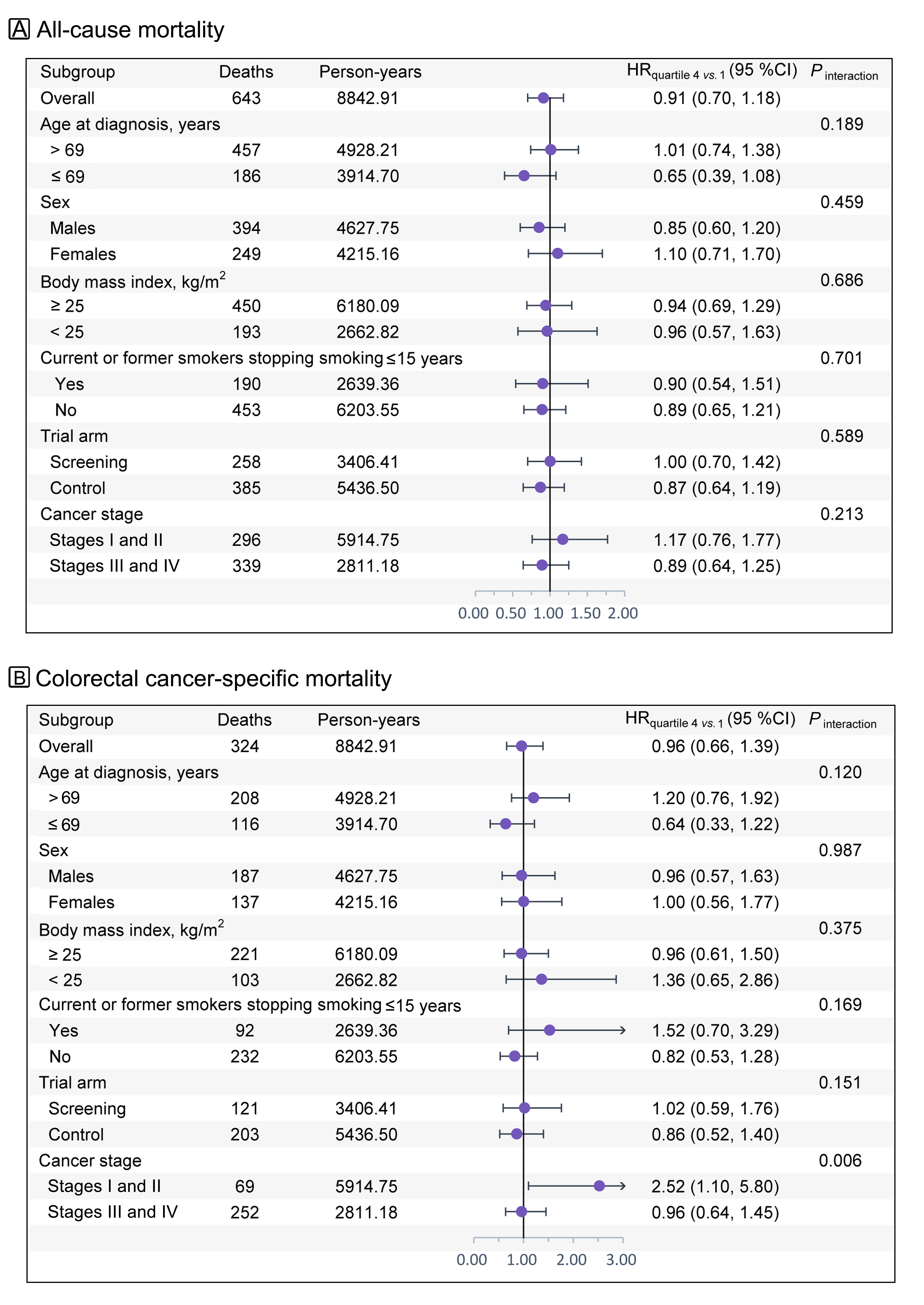


**Figure S4.** Subgroup analyses on the associations of energy-adjusted ultra-processed food consumption before cancer diagnosis with all-cause and colorectal cancer-specific mortality in colorectal cancer patients. Of note, in subgroup analysis by cancer stage, patients with carcinoid cancer were not included.


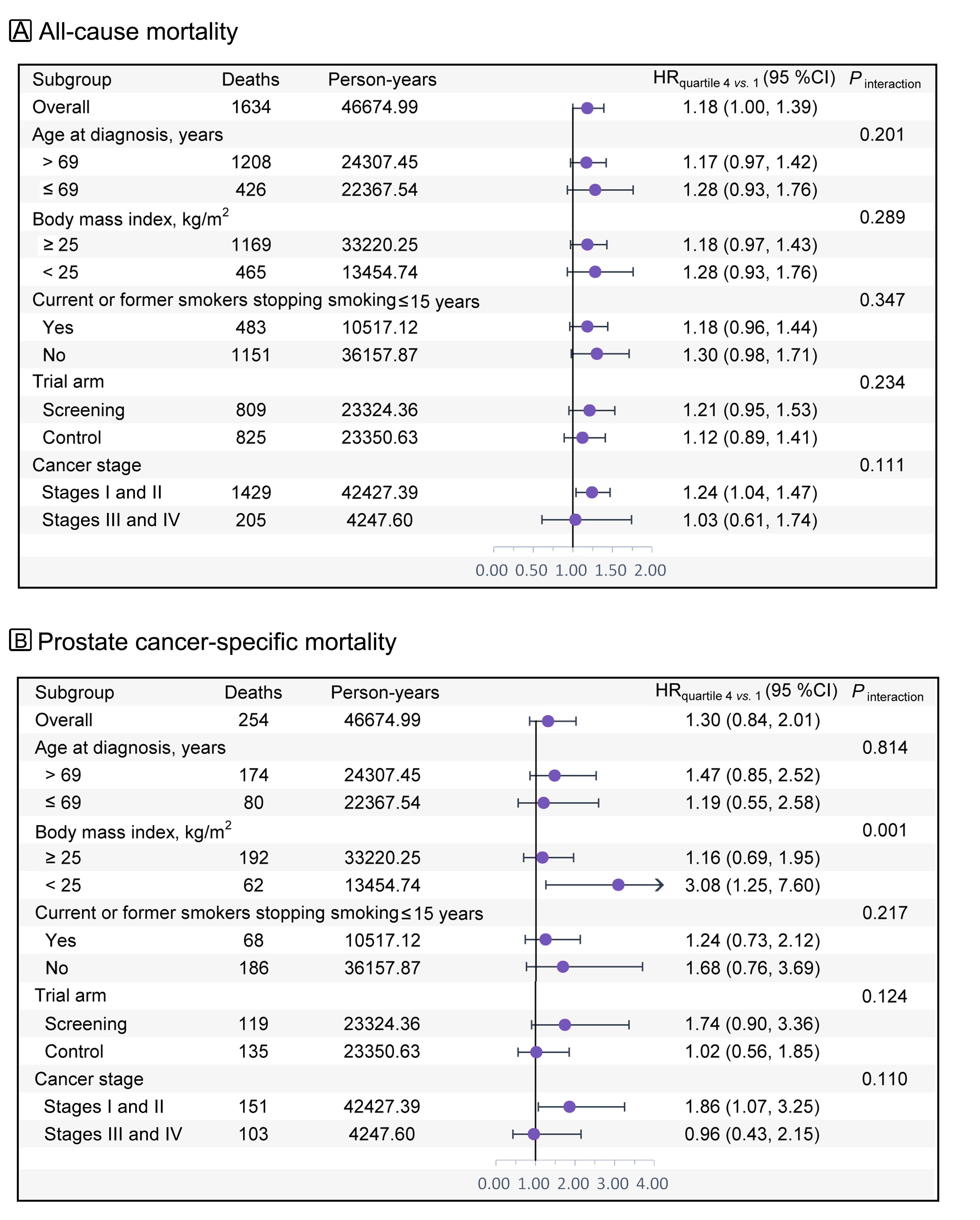


**Figure S5.** Subgroup analyses on the associations of energy-adjusted ultra-processed food consumption before cancer diagnosis with all-cause and prostate cancer-specific mortality in prostate cancer patients.


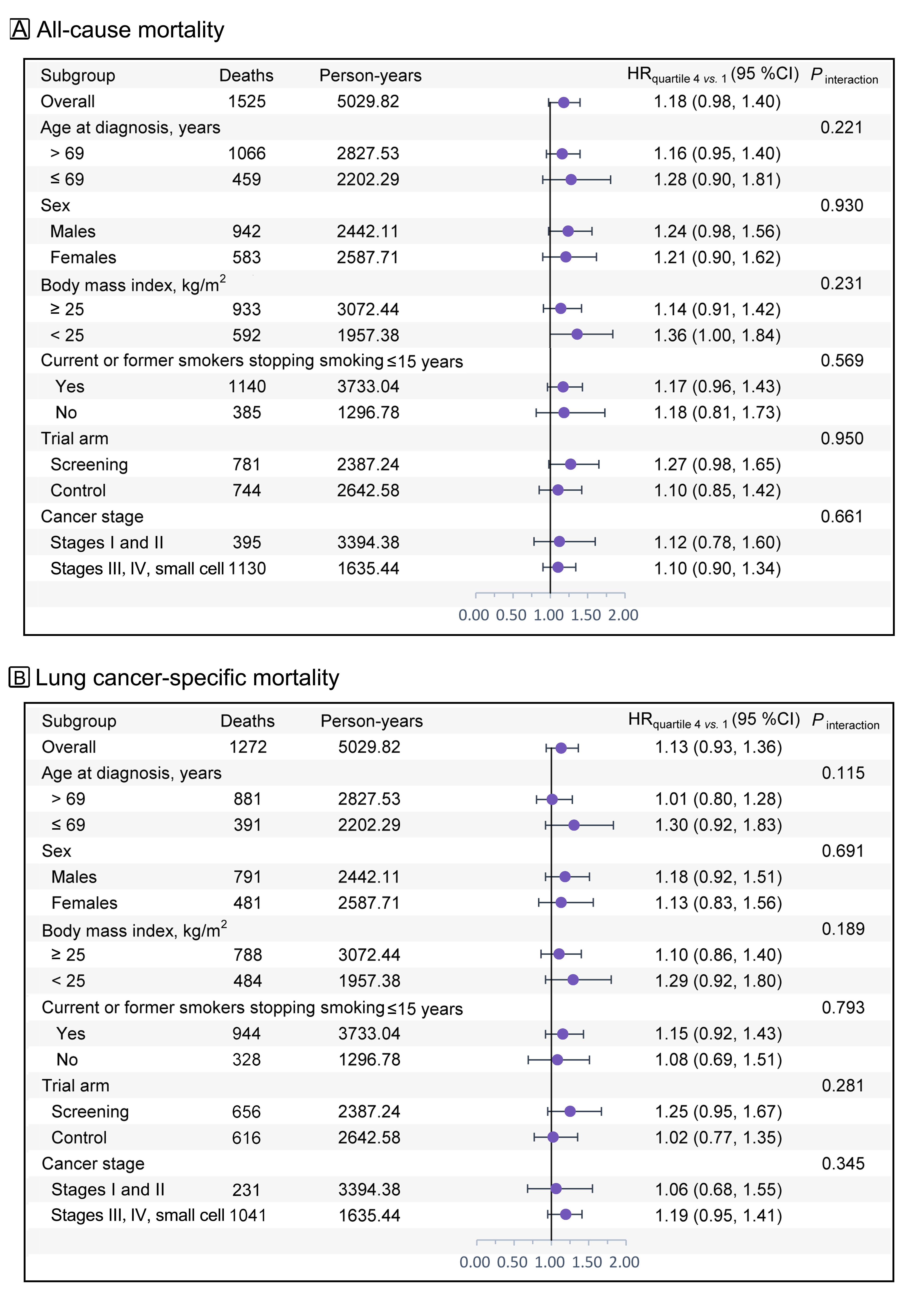


**Figure S6.** Subgroup analyses on the associations of energy-adjusted ultra-processed food consumption before cancer diagnosis with all-cause and lung cancer-specific mortality in lung cancer patients.


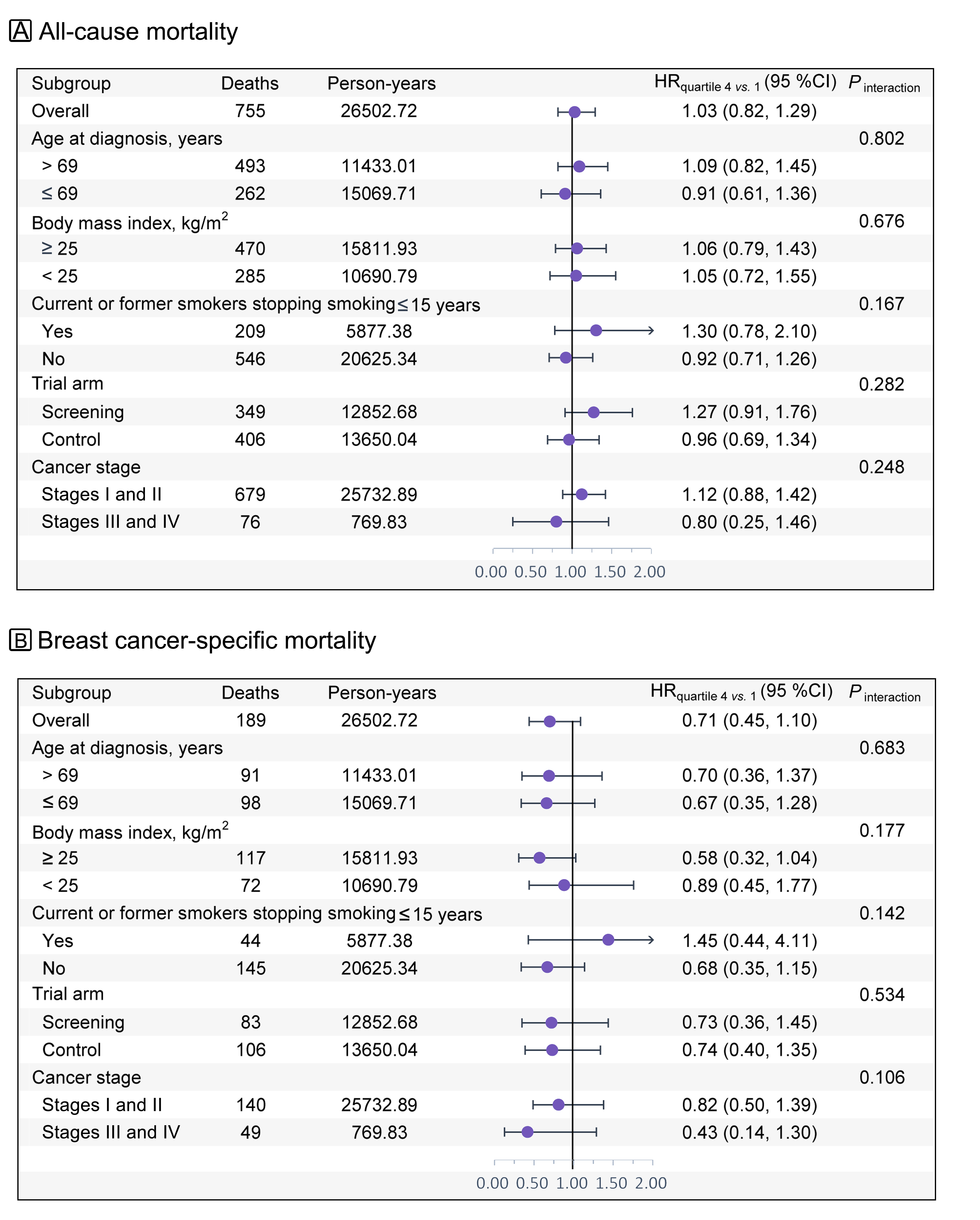


**Figure S7.** Subgroup analyses on the associations of energy-adjusted ultra-processed food consumption before cancer diagnosis with all-cause and breast cancer-specific mortality in breast cancer patients.

**
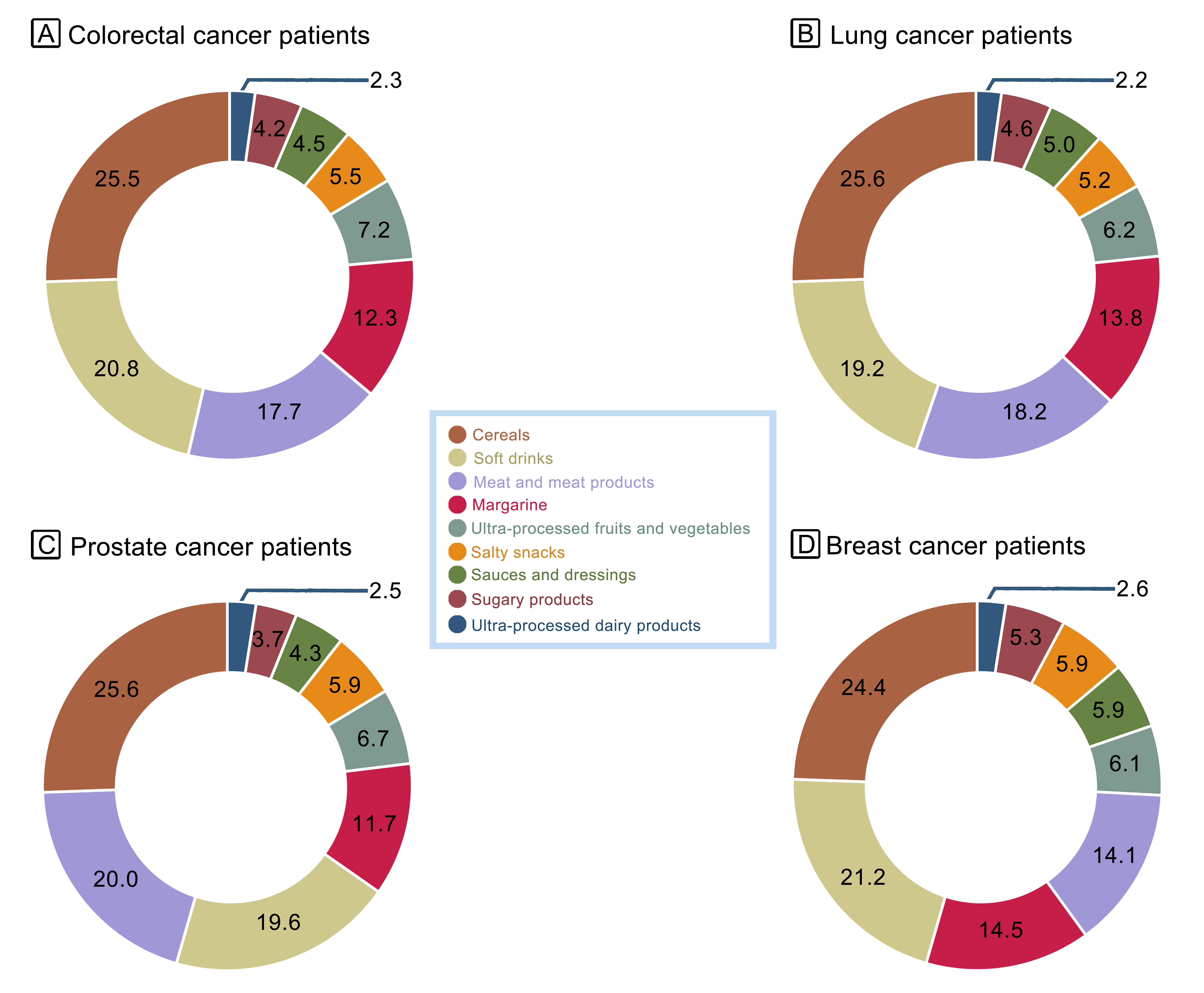
**

**Figure S8.** Proportion (%) of each food subgroup in total energy-unadjusted serving size of ultra-processed foods in four cohorts of cancer patients.

| **Table S1.** Comparison of baseline characteristics between the source and excluded populations ^a^ | | | |
| --- | --- | --- | --- |
| Characteristics | Source population | Excluded population | Standardized  difference ^b^ |
| No. of subjects | 15104 | 14121 |  |
| Age at diagnosis, yrs | 71.2 ± 6.1 | 68.4 ± 6.3 | 0.46 |
| Male, n (%) | 9053 (59.9) | 8725 (61.8) | 0.04 |
| Married, n (%) | 12019 (79.8) | 9927 (74.8) | 0.12 |
| Racial/ethnic group, n (%) | | | |
| Non-Hispanic White | 13900 (92.0) | 11603 (82.2) | 0.31 |
| Non-Hispanic Black | 495 (3.3) | 898 (6.4) |
| Hispanic | 194 (1.3) | 245 (1.7) |
| Other race/ethnicity ^b^ | 515 (3.4) | 1375 (9.7) |
| Occupation, n (%) | | | |
| Homemaker | 1347 (9.0) | 1129 (8.5) | 0.08 |
| Working | 5568 (37.1) | 4644 (35.1) |
| Retired | 7413 (49.3) | 6648 (50.2) |
| Other ^c^ | 695 (4.6) | 817 (6.2) |
| Educational degree, n (%) | | | |
| Some college or less | 9449 (62.7) | 8728 (65.8) | 0.06 |
| College graduate | 2727 (18.1) | 2175 (16.4) |
| Postgraduate | 2893 (19.2) | 2371 (17.9) |
| Body mass index, kg/m^2^ | 27.3 ± 4.6 | 27.2 ± 4.8 | 0.01 |
| Physical activity, min/wk ^d^ | 119.5 ± 122.2 | 116.7 ± 121.1 | 0.02 |
| Alcohol consumption, g/d | 11.4 ± 29.9 | 17.4 ± 70.0 | 0.11 |
| Smoking status, n (%) | | | |
| Current | 1917 (12.7) | 2107 (15.8) | 0.10 |
| Former | 6905 (45.7) | 6188 (46.5) |
| Never | 6279 (41.6) | 5017 (37.7) |
| Pack years | 23.0 ± 30.5 | 26.6 ± 33.4 | 0.11 |
| Years since stopped smoking | 15.7 ± 13.5 | 14.4 ± 13.6 | 0.09 |
| Aspirin user, n (%) | 7228 (48.1) | 6225 (47.2) | 0.02 |
| Family history of cancer, n (%) | 8776 (58.2) | 7646 (57.6) | 0.01 |
| History of diabetes, n (%) | 1041 (6.9) | 1179 (8.9) | 0.07 |
| History of hypertension, n (%) | 5053 (33.6) | 4835 (36.6) | 0.06 |
| Trial arm, n (%) | | | |
| Screening arm | 7410 (49.06) | 7361 (52.13) | 0.06 |
| Control arm | 7694 (50.94) | 6760 (47.87) |
| Energy intake from diet, kcal/d | 1790.0 ± 759.2 | 1859.1 ± 1162.1 | 0.07 |
| Healthy Eating Index-2015 | 65.91 ± 9.76 | 65.67 ± 9.95 | 0.02 |

^a^ Values are mean ± standard deviation or counts (percentage) as indicated.

^b^ The formula used for calculating standardized difference is available in the paper by Austin PC (*Multivariate Behav Res* 2011; 46:399-424).

| **Table S2.** Ultra-processed foods in each food subgroup with assigned serving sizes and energy values | | | |
| --- | --- | --- | --- |
| Food subgroups | Individual ultra-processed foods | No. of servings per 100 g | Energy per 100 g (kcal) |
| Cereals | Quick breads | 2.2 | 297 |
|  | English muffins and bagels | 1.2 | 227 |
|  | Corn bread/muffins | 2.2 | 305 |
|  | Biscuits | 3.0 | 324 |
|  | Pancakes, waffles, and French toast | 1.5 | 270 |
|  | Donuts, sweet rolls, Danishes, and pop tarts | 1.6 | 321 |
|  | White bread/rolls | 1.8 | 266 |
|  | Ready-to-eat cereal, highly fortified | 0.8 | 374 |
|  | Ready-to-eat cereal, other | 0.8 | 374 |
|  | Cookies and brownies | 1.5 | 405 |
|  | Cakes, low fat | 1.7 | 283 |
|  | Cakes, regular | 1.0 | 389 |
|  | Pies, cream/custard/other | 0.6 | 210 |
|  | Pies, fruit | 0.6 | 209 |
|  | Pies, pecan | 0.6 | 407 |
|  | Pies, pumpkin/sweet potato | 1.0 | 260 |
|  | Pizza without meat | 1.5 | 233 |
|  | Macaroni and cheese | 0.3 | 221 |
|  | Lasagna, ravioli, shells | 0.5 | 177 |
| Sauces and dressings | Gravy | 0.1 | 53 |
|  | Cheese sauce | 0.2 | 160 |
|  | Mayonnaise, diet on sandwich | 6.6 | 250 |
|  | Mayonnaise, fat free on sandwich | 1.7 | 64 |
|  | Mayonnaise, regular on sandwich | 6.6 | 680 |
|  | Mayonnaise, diet on salad | 6.6 | 250 |
|  | Mayonnaise, fat free on salad | 1.7 | 64 |
|  | Mayonnaise, regular on salad | 6.6 | 680 |
|  | Salad dressing, low fat on salad and vegetables | 0.3 | 160 |
|  | Salad dressing, nonfat on salad and vegetables | 0.3 | 107 |
|  | Salad dressing, regular on salad and vegetables | 0.3 | 430 |
| Ultra-processed dairy products | Cream cheese, low fat | 0.4 | 201 |
|  | Cream cheese, regular | 0.4 | 350 |
|  | Sour cream, low fat | 0.4 | 181 |
|  | Sour cream, regular | 0.4 | 198 |
|  | Ice cream/ice milk, low fat | 0.4 | 207 |
|  | Regular ice cream | 0.3 | 216 |
|  | Creamed soups | 0.2 | 46 |
|  | Frozen yogurt, ices, and sorbet | 0.4 | 72 |
| Meat and meat products | Fast-food hamburgers | 1.0 | 244 |
|  | Cold cuts | 0.9 | 213 |
|  | Lunch meat | 3.5 | 107 |
|  | Hot dogs, turkey/low fat | 3.0 | 127 |
|  | Hot dogs, regular | 2.7 | 332 |
|  | Sausages | 2.5 | 325 |
|  | Pizza with meat | 0.3 | 277 |
| Soft drinks | Soft drinks, diet/caffeinated | 0.4 | 49 |
|  | Soft drinks, regular/caffeinated | 0.4 | 49 |
|  | Soft drinks, diet/decaffeinated | 0.4 | 40 |
|  | Soft drinks, regular/decaffeinated | 0.4 | 40 |
| Ultra-processed fruits and vegetables | Fruit drinks, diet | 0.3 | 1 |
|  | Fruit drinks, regular | 0.3 | 10 |
|  | Fried potatoes | 1.0 | 196 |
|  | Potato salad | 1.0 | 173 |
| Margarine | Margarine, fat free | 1.5 | 330 |
|  | Margarine, diet | 10 | 533 |
|  | Margarine, diet on bread | 10 | 533 |
|  | Margarine, regular | 10 | 533 |
| Salty snacks | Crackers | 2.1 | 430 |
|  | Potato/corn/other chips | 1.4 | 532 |
|  | Potato/corn/other chips-low fat | 1.4 | 482 |
|  | Popcorn | 3.5 | 530 |
| Sugary products | Candy, chocolate | 2.0 | 462 |
|  | Candy, not chocolate | 0.6 | 394 |
|  | Saccharine in coffee and tea | 23.5 | 360 |

| **Table S3.** Distribution of covariates with missing data before and after imputation in the source population ^a^ | | | |
| --- | --- | --- | --- |
| Covariates | Before imputation | After imputation | Number (%) with missing data |
| History of diabetes, n (%) | | | |
| Yes | 1041 (6.9) | 1041 (6.9) | 79 (0.52) |
| No | 13984 (93.1) | 14063 (93.1) |
| History of hypertension, n (%) | | | |
| Yes | 5053 (33.6) | 5053 (33.5) | 82 (0.54) |
| No | 9969 (66.4) | 10051 (66.5) |
| Family history of colorectal cancer, n (%) | | | |
| Yes | 1559 (10.4) | 1559 (10.3) | 122 (0.81) |
| No | 12982 (86.7) | 13104 (86.8) |
| Possibly ^b^ | 441 (2.9) | 441 (2.9) |
| Family history of lung cancer, n (%) | | | |
| Yes | 1684 (11.2) | 1684 (11.1) | 122 (0.81) |
| No | 12863 (85.9) | 12863 (86.0) |
| Possibly ^b^ | 435 (2.9) | 435 (2.9) |
| Family history of prostate cancer, n (%) | | | |
| Yes | 769 (8.6) | 769 (8.5) | 69 (0.76) |
| No | 8062 (89.7) | 8131 (89.8) |
| Possibly ^b^ | 153 (1.7) | 153 (1.7) |
| Family history of breast cancer, n (%) | | | |
| Yes | 987 (16.5) | 987 (16.3) | 53 (0.88) |
| No | 4946 (82.5) | 4999 (82.6) |
| Male Relative Only | 8 (0.1) | 8 (0.1) |
| Possibly ^b^ | 57 (1.0) | 57 (0.9) |
| Smoking status, n (%) | | | |
| Current | 1917 (12.7) | 1917 (12.7) | 3 (0.02) |
| Former | 6905 (45.7) | 6908 (45.7) |
| Never | 6279 (41.6) | 6279 (41.6) |
| Cigarettes smoked per day | | | |
| >20 | 3735 (24.8) | 3735 (24.7) | 19 (0.13) |
| 10-20 | 3235 (21.4) | 3235 (21.4) |
| <10 | 8115 (53.8) | 8134 (53.9) |
| Years since stopped smoking cigarettes | 15.7 ± 13.5 | 15.7 ± 13.5 | 120 (0.79) |
| Aspirin use, n (%) | | | |
| Yes | 7228 (48.1) | 7228 (47.9) | 83 (0.55) |
| No | 7793 (51.9) | 7876 (52.1) |
| Body mass index, kg/m^2^ | 27.3 ± 4.6 | 27.3 ± 4.6 | 221 (1.46) |
| Physical activity level, min/wk ^c^ | 119.5 ± 122.2 | 113.2 ± 123.3 | 4942 (32.72) |

^a^ Values are mean (standard deviation) or counts (percentage) as indicated.

^b^ “Possibly” means that relative or cancer type is not clear.

^c^ Total time of moderate-to-vigorous physical activity per week.

| **Table S4.** Distribution of clinical covariates with missing data before and after imputation in cancer patients ^a^ | | | |
| --- | --- | --- | --- |
| Covariates | Before imputation | After imputation | Number (%) with missing data |
| **Colorectal cancer patients** | | | |
| Cancer stage ^b^, n (%) | | | |
| Stage I | 324 (29.7) | 332 (30.2) | 8 (0.73) |
| Stage II | 38 (3.5) | 38 (3.5) |
| Stage IIA | 247 (22.6) | 247 (22.5) |
| Stage III | 97 (8.9) | 97 (8.8) |
| Stage IIIA | 34 (3.1) | 34 (3.1) |
| Stage IIIB | 152 (13.9) | 152 (13.8) |
| Stage IIIC | 10 (0.9) | 10 (0.9) |
| Stage IV | 175 (16.0) | 175 (15.9) |
| Carcinoid | 15 (1.4) | 15 (1.4) |
| Surgical resection, n (%) | | | |
| Yes | 1021 (93.0) | 1023 (93.0) | 2 (0.18) |
| No | 77 (7.0) | 77 (7.0) |
| Chemotherapy, n (%) | | | |
| Yes | 486 (44.3) | 486 (44.2) | 2 (0.18) |
| No | 612 (55.7) | 614 (55.8) |
| Radiotherapy, n (%) | | | |
| Yes | 110 (10.0) | 110 (10.0) | 2 (0.18) |
| No | 988 (90.0) | 990 (90.0) |
| **Lung cancer patients** | | | |
| Cancer stage ^b^, n (%) | | | |
| Stage IA | 254 (14.9) | 254 (14.9) | 2 (0.12) |
| Stage IB | 188 (11.0) | 188 (11.0) |
| Stage II | 45 (2.6) | 45 (2.6) |
| Stage IIA | 22 (1.3) | 22 (1.3) |
| Stage IIB | 27 (1.6) | 27 (1.6) |
| Stage III | 10 (0.6) | 10 (0.6) |
| Stage IIIA | 229 (13.4) | 229 (13.4) |
| Stage IIIB | 113 (6.6) | 113 (6.6) |
| Stage IV | 573 (33.6) | 575 (33.7) |
| Small cell | 242 (14.2) | 242 (14.2) |
| Surgical resection, n (%) | | | |
| Yes | 45 (2.7) | 45 (2.6) | 24 (1.41) |
| No | 1636 (97.3) | 1660 (97.4) |
| Chemotherapy, n (%) | | | |
| Yes | 916 (54.5) | 940 (55.1) | 24 (1.41) |
| No | 765 (45.5) | 765 (44.9) |
| Radiotherapy, n (%) | | | |
| Yes | 644 (38.3) | 644 (37.8) | 24 (1.41) |
| No | 1037 (61.7) | 1061 (62.2) |
| **Prostate cancer patients** | | | |
| Cancer stage ^b^, n (%) | | | |
| Stage I | 1493 (34.5) | 1493 (34.4) | 3 (0.07) |
| Stage II | 6 (0.1) | 6 (0.1) |
| Stage IIA | 1912 (44.1) | 1915 (44.2) |
| Stage IIB | 496 (11.4) | 496 (11.4) |
| Stage III | 304 (7.0) | 304 (7.0) |
| Stage IV | 122 (2.8) | 122 (2.8) |
| Gleason score ^c^, n (%) | | | |
| ≤5 | 199 (4.6) | 199 (4.6) | 47 (1.08) |
| 6 | 2043 (47.6) | 2090 (48.2) |
| 7 | 1511 (35.2) | 1511 (34.8) |
| ≥8 | 536 (12.5) | 536 (12.4) |
| PSA level ^d^, ng/mL | 11.4 ± 84.0 | 11.1 ± 81.6 | 251 (5.79) |
| Surgical resection, n (%) | | | |
| Yes | 1380 (32.0) | 1380 (31.8) | 24 (0.55) |
| No | 2932 (68.0) | 2956 (68.2) |
| Radiotherapy, n (%) | | | |
| Yes | 1924 (44.6) | 1924 (44.4) | 24 (0.55) |
| No | 2388 (55.4) | 2412 (55.6) |
| Cryosurgery or hyperthermia therapy, n (%) | | | |
| Yes | 139 (3.2) | 139 (3.2) | 24 (0.55) |
| No | 4173 (96.8) | 4197 (96.8) |
| Hormonal therapy, n (%) | | | |
| Yes | 1449 (33.6) | 1449 (33.4) | 24 (0.55) |
| No | 2863 (66.4) | 2887 (66.6) |
| **Breast cancer patients** | | | |
| Cancer stage ^b^, n (%) | | | |
| Stage 0 | 498 (20.6) | 498 (20.5) | 13 (0.53) |
| Stage I | 314 (13.0) | 314 (12.9) |
| Stage IA | 922 (38.1) | 935 (38.4) |
| Stage IIA | 405 (16.7) | 405 (16.6) |
| Stage IIB | 165 (6.8) | 165 (6.8) |
| Stage III | 2 (0.1) | 2 (0.1) |
| Stage IIIA | 56 (2.3) | 56 (2.3) |
| Stage IIIB | 23 (1.0) | 23 (0.9) |
| Stage IIIC | 7 (0.3) | 7 (0.3) |
| Stage IV | 28 (1.2) | 28 (1.2) |
| Estrogen receptor status, n (%) | | | |
| Positive | 1766 (72.8) | 1772 (72.8) | 6 (0.25) |
| Negative | 317 (13.1) | 317 (13.0) |
| Unknown | 344 (14.2) | 344 (14.1) |
| Progesterone receptor status, n (%) | | | |
| Positive | 1479 (60.9) | 1485 (61.0) | 6 (0.25) |
| Negative | 512 (21.1) | 512 (21.0) |
| Unknown | 436 (18.0) | 436 (17.9) |
| HER2 status, n (%) | | | |
| 0 | 691 (28.5) | 691 (28.4) | 6 (0.25) |
| 1+ | 405 (16.7) | 405 (16.6) |
| 2+ | 227 (9.4) | 227 (9.3) |
| 3+ | 141 (5.8) | 141 (5.8) |
| Unknown | 963 (39.6) | 969 (39.8) |
| Hormone replacement therapy, n (%) | | | |
| Current use | 1380 (56.8) | 1382 (56.8) | 2 (0.08) |
| Former use | 359 (14.8) | 359 (14.8) |
| Never use | 682 (28.1) | 682 (28.0) |
| Unknown | 10 (0.4) | 10 (0.4) |

^a^ Values are mean ± standard deviation or counts (percentage) as indicated.

^b^ Cancer stage was assessed using the AJCC 7th edition staging manual.

^c^ Gleason score was from the prostatectomy if available, otherwise the score was from

the biopsy.

^d^ PSA level was from the most recent test prior to cancer diagnosis.

| **Table S5.** The Akaike’s information criterion (AIC) and Bayesian information criterion (BIC) values for all associations investigated when the number of knots was set at 3, 4, and 5 ^a^ | | | | |
| --- | --- | --- | --- | --- |
| Patients | Associations of interest | 3 knots | 4 knots | 5 knots |
| The AIC value | | | | |
| Colorectal cancer patients | Association with all-cause mortality | 7790.79 | 7792.18 | 7791.95 |
|  | Association with colorectal cancer-specific mortality | 3851.00 | 3851.87 | 3852.39 |
| Lung cancer patients | Association with all-cause mortality | 19094.60 | 19095.84 | 19095.95 |
|  | Association with lung cancer-specific mortality | 16151.44 | 16152.72 | 16154.12 |
| Prostate cancer patients | Association with all-cause mortality | 24238.41 | 24235.71 | 24237.08 |
|  | Association with prostate cancer-specific mortality | 3449.12 | 3450.59 | 3452.48 |
| Breast cancer patients | Association with all-cause mortality | 10488.09 | 10490.55 | 10490.42 |
|  | Association with breast cancer-specific mortality | 2614.56 | 2612.16 | 2617.53 |
| The BIC value | | | | |
| Colorectal cancer patients | Association with all-cause mortality | 7950.80 | 7957.19 | 7961.96 |
|  | Association with colorectal cancer-specific mortality | 4021.01 | 4026.89 | 4032.40 |
| Lung cancer patients | Association with all-cause mortality | 19273.80 | 19280.47 | 19286.00 |
|  | Association with lung cancer-specific mortality | 16325.20 | 16331.92 | 16338.75 |
| Prostate cancer patients | Association with all-cause mortality | 24484.05 | 24480.38 | 24491.79 |
|  | Association with prostate cancer-specific mortality | 3665.62 | 3673.46 | 3681.72 |
| Breast cancer patients | Association with all-cause mortality | 10719.96 | 10734.02 | 10733.89 |
|  | Association with breast cancer-specific mortality | 2846.44 | 2838.24 | 2861.00 |

^a^ The number in bold represents the lowest value in that raw.

| **Table S6.** Cancer characteristics and treatment information of included patients according to quartiles of energy-adjusted ultra-processed food consumption (daily serving) before cancer diagnosis ^a^ | | | | |
| --- | --- | --- | --- | --- |
| Patient group | Quartiles of energy-adjusted ultra-processed food consumption (median, servings/day) | | | |
| **Colorectal cancer patients** | | | | |
|  | Q1 (0.93) | Q2 (2.38) | Q3 (4.21) | Q4 (7.65) |
| Cancer stage ^b^, n (%) | | | | |
| Stage I | 79 (28.7) | 77 (28.0) | 81 (29.5) | 95 (34.5) |
| Stage II | 10 (3.6) | 10 (3.6) | 11 (4.0) | 7 (2.5) |
| Stage IIA | 66 (24.0) | 68 (24.7) | 60 (21.8) | 53 (19.3) |
| Stage III | 24 (8.7) | 16 (5.8) | 26 (9.5) | 31 (11.3) |
| Stage IIIA | 8 (2.9) | 11 (4.0) | 10 (3.6) | 5 (1.8) |
| Stage IIIB | 35 (12.7) | 46 (16.7) | 33 (12.0) | 38 (13.8) |
| Stage IIIC | 4 (1.5) | 3 (1.1) | 2 (0.7) | 1 (0.4) |
| Stage IV | 45 (16.4) | 40 (14.5) | 46 (16.7) | 44 (16.0) |
| Carcinoid | 4 (1.5) | 4 (1.5) | 6 (2.2) | 1 (0.4) |
| Surgical resection, n (%) | | | | |
| Yes | 248 (90.2) | 258 (93.8) | 259 (94.2) | 258 (93.8) |
| No | 27 (9.8) | 17 (6.2) | 16 (5.8) | 17 (6.2) |
| Chemotherapy, n (%) | | | | |
| Yes | 120 (43.6) | 126 (45.8) | 116 (42.2) | 124 (45.1) |
| No | 155 (56.4) | 149 (54.2) | 159 (57.8) | 151 (54.9) |
| Radiotherapy, n (%) | | | | |
| Yes | 21 (7.6) | 37 (13.5) | 25 (9.1) | 27 (9.8) |
| No | 254 (92.4) | 238 (86.5) | 250 (90.9) | 248 (90.2) |
| **Lung cancer patients** | | | | |
|  | Q1 (0.97) | Q2 (2.59) | Q3 (4.55) | Q4 (8.53) |
| Cancer stage ^b^, n (%) | | | | |
| Stage IA | 57 (13.3) | 72 (16.9) | 70 (16.4) | 55 (12.9) |
| Stage IB | 56 (13.1) | 41 (9.6) | 46 (10.8) | 45 (10.6) |
| Stage II | 10 (2.3) | 16 (3.8) | 10 (2.3) | 9 (2.1) |
| Stage IIA | 3 (0.7) | 5 (1.2) | 7 (1.6) | 7 (1.6) |
| Stage IIB | 11 (2.6) | 2 (0.5) | 8 (1.9) | 6 (1.4) |
| Stage III | 1 (0.2) | 2 (0.5) | 4 (0.9) | 3 (0.7) |
| Stage IIIA | 57 (13.3) | 51 (12.0) | 59 (13.8) | 62 (14.6) |
| Stage IIIB | 32 (7.5) | 33 (7.7) | 24 (5.6) | 24 (5.6) |
| Stage IV | 149 (34.9) | 154 (36.2) | 139 (32.6) | 133 (31.2) |
| Small cell | 51 (11.9) | 50 (11.7) | 59 (13.8) | 82 (19.2) |
| Surgical resection, n (%) | | | | |
| Yes | 17 (4.0) | 9 (2.1) | 12 (2.8) | 7 (1.6) |
| No | 410 (96.0) | 417 (97.9) | 414 (97.2) | 419 (98.4) |
| Chemotherapy, n (%) | | | | |
| Yes | 245 (57.4) | 214 (50.2) | 219 (51.4) | 262 (61.5) |
| No | 182 (42.6) | 212 (49.8) | 207 (48.6) | 164 (38.5) |
| Radiotherapy, n (%) | | | | |
| Yes | 163 (38.2) | 147 (34.5) | 168 (39.4) | 166 (39.0) |
| No | 264 (61.8) | 279 (65.5) | 258 (60.6) | 260 (61.0) |
| **Prostate cancer patients** | | | | |
|  | Q1 (1.14) | Q2 (2.85) | Q3 (4.77) | Q4 (8.52) |
| Cancer stage ^b^, n (%) | | | | |
| Stage I | 377 (34.8) | 377 (34.8) | 351 (32.4) | 388 (35.8) |
| Stage II | 2 (0.2) | 0 (0.0) | 3 (0.3) | 1 (0.1) |
| Stage IIA | 468 (43.2) | 480 (44.3) | 494 (45.6) | 473 (43.6) |
| Stage IIB | 118 (10.9) | 135 (12.5) | 132 (12.2) | 111 (10.2) |
| Stage III | 81 (7.5) | 65 (6.0) | 75 (6.9) | 83 (7.7) |
| Stage IV | 38 (3.5) | 27 (2.5) | 29 (2.7) | 28 (2.6) |
| Gleason score ^c^, n (%) | | | | |
| ≤5 | 49 (4.5) | 55 (5.1) | 47 (4.3) | 48 (4.4) |
| 6 | 526 (48.5) | 505 (46.6) | 512 (47.2) | 547 (50.5) |
| 7 | 382 (35.2) | 389 (35.9) | 381 (35.1) | 359 (33.1) |
| ≥8 | 127 (11.7) | 135 (12.5) | 144 (13.3) | 130 (12.0) |
| PSA level ^d^, ng/mL | 11.1 ± 39.4 | 10.2 ± 44.2 | 8.7 ± 16.8 | 14.2 ± 151.2 |
| Surgical resection, n (%) | | | | |
| Yes | 311 (28.7) | 333 (30.7) | 372 (34.3) | 364 (33.6) |
| No | 773 (71.3) | 751 (69.3) | 712 (65.7) | 720 (66.4) |
| Radiotherapy, n (%) | | | | |
| Yes | 489 (45.1) | 485 (44.7) | 457 (42.2) | 493 (45.5) |
| No | 595 (54.9) | 599 (55.3) | 627 (57.8) | 591 (54.5) |
| Cryosurgery or hyperthermia therapy, n (%) | | | | |
| Yes | 36 (3.3) | 33 (3.0) | 36 (3.3) | 34 (3.1) |
| No | 1048 (96.7) | 1051 (97.0) | 1048 (96.7) | 1050 (96.9) |
| Hormonal therapy, n (%) | | | | |
| Yes | 364 (33.6) | 372 (34.3) | 358 (33.0) | 355 (32.7) |
| No | 720 (66.4) | 712 (65.7) | 726 (67.0) | 729 (67.3) |
| **Breast cancer patients** | | | | |
|  | Q1 (0.63) | Q2 (1.98) | Q3 (3.60) | Q4 (6.72) |
| Cancer stage ^b^, n (%) | | | | |
| Stage 0 | 123 (20.2) | 130 (21.4) | 137 (22.5) | 108 (17.8) |
| Stage I | 86 (14.1) | 61 (10.0) | 94 (15.5) | 73 (12.0) |
| Stage IA | 234 (38.4 | 247 (40.6) | 231 (38.0) | 223 (36.7) |
| Stage IIA | 100 (16.4) | 111 (18.3) | 87 (14.3) | 107 (17.6) |
| Stage IIB | 40 (6.6) | 40 (6.6) | 28 (4.6) | 57 (9.4) |
| Stage III | 2 (0.3) | 0 (0.0) | 0 (0.0) | 0 (0.0) |
| Stage IIIA | 13 (2.1) | 8 (1.3) | 11 (1.8) | 24 (3.9) |
| Stage IIIB | 3 (0.5) | 7 (1.2) | 5 (0.8) | 8 (1.3) |
| Stage IIIC | 2 (0.3) | 1 (0.2) | 3 (0.5) | 1 (0.2) |
| Stage IV | 6 (1.0) | 3 (0.5) | 12 (2.0) | 7 (1.2) |
| Estrogen receptor status, n (%) | | | | |
| Positive | 452 (74.2) | 436 (71.7) | 440 (72.4) | 444 (73.0) |
| Negative | 69 (11.3) | 84 (13.8) | 81 (13.3) | 83 (13.7) |
| Unknown | 88 (14.4) | 88 (14.5) | 87 (14.3) | 81 (13.3) |
| Progesterone receptor status, n (%) | | | | |
| Positive | 381 (62.6) | 369 (60.7) | 356 (58.6) | 379 (62.3) |
| Negative | 114 (18.7) | 130 (21.4) | 142 (23.4) | 126 (20.7) |
| Unknown | 114 (18.7) | 109 (17.9) | 110 (18.1) | 103 (16.9) |
| HER2 status, n (%) | | | | |
| 0 | 184 (30.2) | 163 (26.8) | 171 (28.1) | 173 (28.5) |
| 1+ | 100 (16.4) | 100 (16.4) | 107 (17.6) | 98 (16.1) |
| 2+ | 54 (8.9) | 62 (10.2) | 47 (7.7) | 64 (10.5) |
| 3+ | 28 (4.6) | 33 (5.4) | 33 (5.4) | 47 (7.7) |
| Unknown | 243 (39.9) | 250 (41.1) | 250 (41.1) | 226 (37.2) |
| Hormone replacement therapy, n (%) | | | | |
| Current use | 358 (58.8) | 355 (58.4) | 341 (56.1) | 328 (53.9) |
| Former use | 97 (15.9) | 80 (13.2) | 90 (14.8) | 92 (15.1) |
| Never use | 153 (25.1) | 171 (28.1) | 175 (28.8) | 183 (30.1) |
| Unknown | 1 (0.2) | 2 (0.3) | 2 (0.3) | 5 (0.8) |

Abbreviation: quartile, Q.

^a^ Values are mean ± standard deviation or counts (percentage) as indicated.

^b^ Cancer stage was assessed using the AJCC 7th edition staging manual.

^c^ Gleason score was from the prostatectomy if available, otherwise the score was from

the biopsy.

^d^ PSA level was from the most recent test prior to cancer diagnosis.

| **Table S7.** Hazard ratios (95% confidence interval) for associations of energy-adjusted ultra-processed food consumption (daily gram) before cancer diagnosis with all-cause and cancer-specific mortality in patients with colorectal, lung, prostate, or breast cancer | | | | | |
| --- | --- | --- | --- | --- | --- |
| Patient group | Quartiles of energy-adjusted ultra-processed food consumption (median, grams/day) | | | | *P*_trend_ |
| **Colorectal cancer patients** | | | | | |
|  | Q1 (138.48) | Q2 (252.99) | Q3 (433.39) | Q4 (826.75) |  |
| *All-cause mortality* | | | | | |
| Model 1 ^a^ | 1.00 (reference) | 1.04 (0.83, 1.29) | 0.96 (0.77, 1.21) | 1.03 (0.82, 1.31) | 0.879 |
| Model 2 ^b^ | 1.00 (reference) | 1.04 (0.82, 1.30) | 1.06 (0.83, 1.35) | 1.04 (0.79, 1.37) | 0.856 |
| *Colorectal cancer-specific mortality* | | | | | |
| Model 1 ^a^ | 1.00 (reference) | 0.88 (0.65, 1.20) | 0.80 (0.58, 1.10) | 0.89 (0.65, 1.23) | 0.628 |
| Model 2 ^b^ | 1.00 (reference) | 0.95 (0.69, 1.31) | 0.94 (0.67, 1.33) | 1.01 (0.69, 1.46) | 0.884 |
| **Lung cancer patients** | | | | | |
|  | Q1 (138.80) | Q2 (252.96) | Q3 (417.67) | Q4 (836.78) |  |
| *All-cause mortality* | | | | | |
| Model 1 ^a^ | 1.00 (reference) | 0.98 (0.84, 1.13) | 0.95 (0.82, 1.10) | 1.19 (1.03, 1.38) | 0.004 |
| Model 2 ^b^ | 1.00 (reference) | 1.01 (0.87, 1.17) | 0.97 (0.83, 1.14) | 1.24 (1.04, 1.48) | 0.004 |
| *Lung cancer-specific mortality* | | | | | |
| Model 1 ^a^ | 1.00 (reference) | 0.96 (0.81, 1.12) | 0.92 (0.79, 1.08) | 1.14 (0.97, 1.34) | 0.033 |
| Model 2 ^b^ | 1.00 (reference) | 1.00 (0.84, 1.18) | 0.93 (0.79, 1.11) | 1.14 (0.94, 1.37) | 0.113 |
| **Prostate cancer patients** | | | | | |
|  | Q1 (171.46) | Q2 (298.58) | Q3 (471.84) | Q4 (895.34) |  |
| *All-cause mortality* | | | | | |
| Model 1 ^a^ | 1.00 (reference) | 0.97 (0.84, 1.11) | 1.15 (1.01, 1.32) | 1.21 (1.05, 1.39) | 0.001 |
| Model 2 ^b^ | 1.00 (reference) | 0.95 (0.82, 1.10) | 1.11 (0.96, 1.28) | 1.14 (0.96, 1.34) | 0.048 |
| *Prostate cancer-specific mortality* | | | | | |
| Model 1 ^a^ | 1.00 (reference) | 0.89 (0.62, 1.28) | 1.29 (0.92, 1.81) | 1.14 (0.79, 1.63) | 0.260 |
| Model 2 ^b^ | 1.00 (reference) | 0.78 (0.53, 1.14) | 1.12 (0.77, 1.63) | 1.04 (0.67, 1.62) | 0.511 |
| **Breast cancer patients** | | | | | |
|  | Q1 (116.74) | Q2 (210.49) | Q3 (346.06) | Q4 (731.48) |  |
| *All-cause mortality* | | | | | |
| Model 1 ^a^ | 1.00 (reference) | 1.02 (0.83, 1.25) | 1.12 (0.92, 1.37) | 1.19 (0.97, 1.46) | 0.065 |
| Model 2 ^b^ | 1.00 (reference) | 0.91 (0.74, 1.13) | 1.04 (0.84, 1.29) | 0.97 (0.77, 1.23) | 0.969 |
| *Breast cancer-specific mortality* | | | | | |
| Model 1 ^a^ | 1.00 (reference) | 0.89 (0.59, 1.38) | 1.09 (0.73, 1.63) | 1.19 (0.80, 1.77) | 0.241 |
| Model 2 ^b^ | 1.00 (reference | 0.69 (0.44, 1.09) | 0.89 (0.57, 1.40) | 0.81 (0.51, 1.29) | 0.765 |

Abbreviation: quartile, Q.

^a^ Adjusted for age at diagnosis (years), sex (male, female; only for colorectal and lung cancers), and racial/ethnic group (non-Hispanic White, non-Hispanic Black, Hispanic, others).

^b^ Adjusted for model 1 plus trial arm (screening, control), body mass index (kg/m^2^), physical activity (min/week), alcohol consumption (g/day), smoking status [current (>20 cigarettes/day, 10-20 cigarettes/day, <10 cigarettes/day), former (stop smoking >15 years, stop smoking ≤15 years), never], aspirin use (yes, no), energy intake from diet (kcal/day), family history of indicated cancer (yes, no), history of diabetes (yes, no), history of hypertension (yes, no; only for all-cause mortality), and clinical covariates. For colorectal cancer, clinical covariates were cancer stage (9 categories), surgical resection (yes, no), chemotherapy (yes, no), and radiotherapy (yes, no); for lung cancer, clinical covariates were cancer stage (11 categories), surgical resection (yes, no), chemotherapy (yes, no), and radiotherapy (yes, no); for prostate cancer, clinical covariates were cancer stage (6 categories), Gleason score (2 to 10 points), PSA level closest to diagnosis (ng/mL), surgical resection (yes, no), radiotherapy (yes, no), cryosurgery or hyperthermia therapy (yes, no), and hormonal therapy (yes, no); for breast cancer, clinical covariates were cancer stage (10 categories), estrogen receptor status (positive, negative, unknown), progesterone receptor status (positive, negative, unknown), HER2 status (0, 1+, 2+, 3+, unknown), and hormone replacement therapy (current use, former use, never use, unknown).

| **Table S8.** Hazard ratios (95% confidence interval) for associations of energy-adjusted ultra-processed food consumption (daily serving/kilogram body weight) before cancer diagnosis with all-cause and cancer-specific mortality in patients with colorectal, lung, prostate, or breast cancer | | | | | |
| --- | --- | --- | --- | --- | --- |
| Patient group | Quartiles of energy-adjusted ultra-processed food consumption (median, daily serving/kilogram body weight) | | | | *P*_trend_ |
| **Colorectal cancer patients** | | | | | |
|  | Q1 (-0.80) | Q2 (-0.34) | Q3 (0.11) | Q4 (1.04) |  |
| *All-cause mortality* | | | | | |
| Model 1 ^a^ | 1.00 (reference) | 1.00 (0.81, 1.24) | 0.96 (0.77, 1.19) | 0.94 (0.75, 1.17) | 0.529 |
| Model 2 ^b^ | 1.00 (reference) | 1.14 (0.91, 1.43) | 0.94 (0.75, 1.19) | 0.91 (0.72, 1.16) | 0.250 |
| *Colorectal cancer-specific mortality* | | | | | |
| Model 1 ^a^ | 1.00 (reference) | 1.12 (0.82, 1.52) | 1.05 (0.76, 1.44) | 0.96 (0.70, 1.33) | 0.642 |
| Model 2 ^b^ | 1.00 (reference) | 1.39 (1.00, 1.94) | 1.07 (0.77, 1.49) | 0.96 (0.68, 1.35) | 0.420 |
| **Lung cancer patients** | | | | | |
|  | Q1 (-0.77) | Q2 (-0.34) | Q3 (0.10) | Q4 (1.02) |  |
| *All-cause mortality* | | | | | |
| Model 1 ^a^ | 1.00 (reference) | 1.21 (1.05, 1.40) | 1.10 (0.95, 1.27) | 1.15 (1.00, 1.33) | 0.191 |
| Model 2 ^b^ | 1.00 (reference) | 1.24 (1.07, 1.45) | 1.10 (0.95, 1.29) | 1.21 (1.04, 1.42) | 0.084 |
| *Lung cancer-specific mortality* | | | | | |
| Model 1 ^a^ | 1.00 (reference) | 1.17 (1.00, 1.36) | 1.03 (0.88, 1.21) | 1.09 (0.93, 1.27) | 0.604 |
| Model 2 ^b^ | 1.00 (reference) | 1.19 (1.01, 1.40) | 1.03 (0.87, 1.22) | 1.13 (0.95, 1.34) | 0.382 |
| **Prostate cancer patients** | | | | | |
|  | Q1 (-0.83) | Q2 (-0.32) | Q3 (0.13) | Q4 (1.01) |  |
| *All-cause mortality* | | | | | |
| Model 1 ^a^ | 1.00 (reference) | 0.93 (0.80, 1.06) | 1.10 (0.96, 1.26) | 1.13 (0.98, 1.30) | 0.017 |
| Model 2 ^b^ | 1.00 (reference) | 0.92 (0.79, 1.06) | 1.15 (1.00, 1.32) | 1.14 (0.99, 1.32) | 0.011 |
| *Prostate cancer-specific mortality* | | | | | |
| Model 1 ^a^ | 1.00 (reference) | 0.89 (0.63, 1.26) | 0.95 (0.67, 1.34) | 1.08 (0.76, 1.52) | 0.540 |
| Model 2 ^b^ | 1.00 (reference) | 0.66 (0.45, 0.97) | 0.97 (0.67, 1.39) | 1.06 (0.73, 1.54) | 0.280 |
| **Breast cancer patients** | | | | | |
|  | Q1 (-0.88) | Q2 (-0.37) | Q3 (0.15) | Q4 (1.04) |  |
| *All-cause mortality* | | | | | |
| Model 1 ^a^ | 1.00 (reference) | 1.00 (0.81, 1.22) | 1.11 (0.90, 1.36) | 1.15 (0.94, 1.41) | 0.109 |
| Model 2 ^b^ | 1.00 (reference) | 1.03 (0.83, 1.28) | 1.05 (0.85, 1.30) | 1.02 (0.82, 1.27) | 0.793 |
| *Breast cancer-specific mortality* | | | | | |
| Model 1 ^a^ | 1.00 (reference) | 0.83 (0.55, 1.26) | 1.25 (0.85, 1.83) | 0.82 (0.54, 1.25) | 0.829 |
| Model 2 ^b^ | 1.00 (reference | 1.01 (0.65, 1.56) | 1.07 (0.71, 1.59) | 0.72 (0.47, 1.12) | 0.209 |

Abbreviation: quartile, Q.

^a^ Adjusted for age at diagnosis (years), sex (male, female; only for colorectal and lung cancers), and racial/ethnic group (non-Hispanic White, non-Hispanic Black, Hispanic, others).

^b^ Adjusted for model 1 plus trial arm (screening, control), body mass index (kg/m^2^), physical activity (min/week), alcohol consumption (g/day), smoking status [current (>20 cigarettes/day, 10-20 cigarettes/day, <10 cigarettes/day), former (stop smoking >15 years, stop smoking ≤15 years), never], aspirin use (yes, no), energy intake from diet (kcal/day), family history of indicated cancer (yes, no), history of diabetes (yes, no), history of hypertension (yes, no; only for all-cause mortality), and clinical covariates. For colorectal cancer, clinical covariates were cancer stage (9 categories), surgical resection (yes, no), chemotherapy (yes, no), and radiotherapy (yes, no); for lung cancer, clinical covariates were cancer stage (11 categories), surgical resection (yes, no), chemotherapy (yes, no), and radiotherapy (yes, no); for prostate cancer, clinical covariates were cancer stage (6 categories), Gleason score (2 to 10 points), PSA level closest to diagnosis (ng/mL), surgical resection (yes, no), radiotherapy (yes, no), cryosurgery or hyperthermia therapy (yes, no), and hormonal therapy (yes, no); for breast cancer, clinical covariates were cancer stage (10 categories), estrogen receptor status (positive, negative, unknown), progesterone receptor status (positive, negative, unknown), HER2 status (0, 1+, 2+, 3+, unknown), and hormone replacement therapy (current use, former use, never use, unknown).

| **Table S9.** Subdistribution hazard ratios (95% confidence interval) for the association of energy-adjusted ultra-processed food consumption (daily serving) before cancer diagnosis with cancer-specific mortality in patients with colorectal, lung, prostate, or breast cancer | | | | | |
| --- | --- | --- | --- | --- | --- |
| Cancer-specific mortality | Quartiles of energy-adjusted ultra-processed food consumption before cancer diagnosis (median, servings/day) | | | | *P*_trend_ |
| Colorectal cancer-specific mortality in colorectal cancer patients | | | | | |
|  | Q1 (0.93) | Q2 (2.38) | Q3 (4.21) | Q4 (7.65) |  |
| Model 1 ^a^ | 1.00 (reference) | 0.95 (0.70, 1.30) | 0.87 (0.63, 1.19) | 0.92 (0.67, 1.27) | 0.618 |
| Model 2 ^b^ | 1.00 (reference) | 1.01 (0.73, 1.42) | 0.91 (0.65, 1.27) | 0.93 (0.64, 1.35) | 0.605 |
| Lung cancer-specific mortality in lung cancer patients | | | | | |
|  | Q1 (0.97) | Q2 (2.59) | Q3 (4.55) | Q4 (8.53) |  |
| Model 1 ^a^ | 1.00 (reference) | 1.04 (0.88, 1.21) | 1.04 (0.89, 1.21) | 1.08 (0.92, 1.26) | 0.395 |
| Model 2 ^b^ | 1.00 (reference) | 1.04 (0.86, 1.26) | 1.16 (0.97, 1.38) | 1.08 (0.89, 1.33) | 0.421 |
| Prostate cancer-specific mortality in prostate cancer patients | | | | | |
|  | Q1 (1.14) | Q2 (2.85) | Q3 (4.77) | Q4 (8.52) |  |
| Model 1 ^a^ | 1.00 (reference) | 1.01 (0.70, 1.45) | 1.36 (0.96, 1.92) | 1.28 (0.89, 1.83) | 0.099 |
| Model 2 ^b^ | 1.00 (reference) | 0.98 (0.65, 1.46) | 1.25 (0.84, 1.86) | 1.24 (0.77, 1.97) | 0.269 |
| Breast cancer-specific mortality in breast cancer patients | | | | | |
|  | Q1 (0.63) | Q2 (1.98) | Q3 (3.60) | Q4 (6.72) |  |
| Model 1 ^a^ | 1.00 (reference) | 0.78 (0.50, 1.18) | 0.89 (0.60, 1.34) | 1.00 (0.68, 1.49) | 0.671 |
| Model 2 ^b^ | 1.00 (reference | 0.75 (0.48, 1.16) | 0.72 (0.46, 1.12) | 0.67 (0.44, 1.04) | 0.117 |

Abbreviation: quartile, Q.

^a^ Adjusted for age at diagnosis (years), sex (male, female; only for colorectal and lung cancers), and racial/ethnic group (non-Hispanic White, non-Hispanic Black, Hispanic, others).

^b^ Adjusted for model 1 plus trial arm (screening, control), body mass index (kg/m^2^), physical activity (min/week), alcohol consumption (g/day), smoking status [current (>20 cigarettes/day, 10-20 cigarettes/day, <10 cigarettes/day), former (stop smoking >15 years, stop smoking ≤15 years), never], aspirin use (yes, no), energy intake from diet (kcal/day), family history of indicated cancer (yes, no), history of diabetes (yes, no), and clinical covariates. For colorectal cancer, clinical covariates were cancer stage (9 categories), surgical resection (yes, no), chemotherapy (yes, no), and radiotherapy (yes, no); for lung cancer, clinical covariates were cancer stage (11 categories), surgical resection (yes, no), chemotherapy (yes, no), and radiotherapy (yes, no); for prostate cancer, clinical covariates were cancer stage (6 categories), Gleason score (2 to 10 points), PSA level closest to diagnosis (ng/mL), surgical resection (yes, no), radiotherapy (yes, no), cryosurgery or hyperthermia therapy (yes, no), and hormonal therapy (yes, no); for breast cancer, clinical covariates were cancer stage (10 categories), estrogen receptor status (positive, negative, unknown), progesterone receptor status (positive, negative, unknown), HER2 status (0, 1+, 2+, 3+, unknown), and hormone replacement therapy (current use, former use, never use, unknown).

| **Table S10.** Sensitivity analyses on the associations of energy-adjusted ultra-processed food consumption (daily serving) before cancer diagnosis with all-cause and cancer-specific mortality in patients with colorectal, lung, prostate, or breast cancer ^a^ | | | | | | |
| --- | --- | --- | --- | --- | --- | --- |
| Patient group | Quartiles of energy-adjusted ultra-processed food consumption (median, servings/day) | | | | | |
| **Colorectal cancer patients** | | | | | | |
|  | *All-cause mortality* | | | *Colorectal cancer-specific mortality* | | |
|  | Q1 (0.93) | Q4 (7.65) | *P*_trend_ | Q1 (0.93) | Q4 (7.65) | *P*_trend_ |
| Excluded patients whose colorectal cancer was not the first diagnosed cancer (n=72) | 1.00 (reference) | 0.92 (0.70, 1.21) | 0.350 | 1.00 (reference) | 0.94 (0.64, 1.38) | 0.576 |
| Excluded patients whose colorectal cancer was diagnosed ≤2 years after DHQ completion (n=242) | 1.00 (reference) | 0.89 (0.65, 1.20) | 0.191 | 1.00 (reference) | 0.92 (0.60, 1.41) | 0.447 |
| Excluded patients who died within 30 days after colorectal cancer diagnosis (n=27) | 1.00 (reference) | 0.87 (0.67, 1.14) | 0.155 | 1.00 (reference) | 0.90 (0.61, 1.32) | 0.397 |
| Excluded patients who died within 90 days after colorectal cancer diagnosis (n=58) | 1.00 (reference) | 0.86 (0.65, 1.13) | 0.100 | 1.00 (reference) | 0.87 (0.58, 1.30) | 0.230 |
| Excluded patients with extreme ultra-processed food consumption (n=55) ^b^ | 1.00 (reference) | 0.87 (0.66, 1.15) | 0.195 | 1.00 (reference) | 0.89 (0.61, 1.31) | 0.414 |
| Excluded patients with extreme energy intake (n=40) ^c^ | 1.00 (reference) | 0.93 (0.71, 1.21) | 0.411 | 1.00 (reference) | 1.02 (0.69, 1.50) | 0.837 |
| Repeated analysis with sex-specific quartiles | 1.00 (reference) | 0.88 (0.68, 1.14) | 0.176 | 1.00 (reference) | 0.99 (0.69, 1.41) | 0.623 |
| Additional adjustment on model 2 ^d^ | | | | | | |
| Intakes of fruit, vegetable, coffee, dairy, fish, whole grain, and red and processed meat | 1.00 (reference) | 0.94 (0.71, 1.26) | 0.430 | 1.00 (reference) | 0.96 (0.64, 1.44) | 0.621 |
| Intakes of dietary fiber, added sugar, saturated fatty acids, and polyunsaturated fatty acids | 1.00 (reference) | 0.94 (0.71, 1.25) | 0.462 | 1.00 (reference) | 0.99 (0.66, 1.49) | 0.747 |
| Healthy Eating Index-2015 | 1.00 (reference) | 0.85 (0.65, 1.12) | 0.131 | 1.00 (reference) | 0.89 (0.61, 1.31) | 0.377 |
| Western diet score | 1.00 (reference) | 0.92 (0.70, 1.20) | 0.328 | 1.00 (reference) | 0.93 (0.64, 1.37) | 0.538 |
| Glycemic index | 1.00 (reference) | 0.96 (0.72, 1.27) | 0.550 | 1.00 (reference) | 0.95 (0.64, 1.41) | 0.608 |
| Glycemic load | 1.00 (reference) | 0.89 (0.68, 1.16) | 0.221 | 1.00 (reference) | 0.95 (0.66, 1.38) | 0.578 |
| **Lung cancer patients** | | | | | | |
|  | *All-cause mortality* | | | *Lung cancer-specific mortality* | | |
|  | Q1 (0.97) | Q4 (8.53) | *P*_trend_ | Q1 (0.97) | Q4 (8.53) | *P*_trend_ |
| Excluded patients whose lung cancer was not the first diagnosed cancer (n=174) | 1.00 (reference) | 1.17 (0.97, 1.41) | 0.024 | 1.00 (reference) | 1.10 (0.90, 1.35) | 0.179 |
| Excluded patients whose colorectal cancer was diagnosed ≤2 years after DHQ completion (n=308) | 1.00 (reference) | 1.17 (0.96, 1.42) | 0.079 | 1.00 (reference) | 1.07 (0.86, 1.33) | 0.465 |
| Excluded patients who died within 30 days after lung cancer diagnosis (n=172) | 1.00 (reference) | 1.20 (0.99, 1.45) | 0.010 | 1.00 (reference) | 1.13 (0.92, 1.39) | 0.103 |
| Excluded patients who died within 90 days after lung cancer diagnosis (n=378) | 1.00 (reference) | 1.21 (0.98, 1.48) | 0.009 | 1.00 (reference) | 1.13 (0.90, 1.42) | 0.090 |
| Excluded patients with extreme ultra-processed food consumption (n=86) ^b^ | 1.00 (reference) | 1.15 (0.95, 1.38) | 0.042 | 1.00 (reference) | 1.10 (0.90, 1.34) | 0.215 |
| Excluded patients with extreme energy intake (n=64) ^c^ | 1.00 (reference) | 1.16 (0.97, 1.39) | 0.039 | 1.00 (reference) | 1.11 (0.91, 1.35) | 0.209 |
| Repeated analysis with sex-specific quartiles | 1.00 (reference) | 1.17 (0.99, 1.40) | 0.065 | 1.00 (reference) | 1.09 (0.91, 1.32) | 0.305 |
| Additional adjustment on model 2 ^d^ | | | | | | |
| Intakes of fruit, vegetable, coffee, dairy, fish, whole grain, and red and processed meat | 1.00 (reference) | 1.22 (1.01, 1.47) | 0.010 | 1.00 (reference) | 1.11 (0.92, 1.34) | 0.116 |
| Intakes of dietary fiber, added sugar, saturated fatty acids, and polyunsaturated fatty acids | 1.00 (reference) | 1.18 (0.96, 1.44) | 0.063 | 1.00 (reference) | 1.08 (0.88, 1.32) | 0.337 |
| Healthy Eating Index-2015 | 1.00 (reference) | 1.13 (0.94, 1.37) | 0.066 | 1.00 (reference) | 1.10 (0.90, 1.35) | 0.237 |
| Western diet score | 1.00 (reference) | 1.14 (0.95, 1.36) | 0.048 | 1.00 (reference) | 1.09 (0.90, 1.32) | 0.250 |
| Glycemic index | 1.00 (reference) | 1.13 (0.93, 1.38) | 0.069 | 1.00 (reference) | 1.07 (0.87, 1.32) | 0.358 |
| Glycemic load | 1.00 (reference) | 1.17 (0.97, 1.39) | 0.029 | 1.00 (reference) | 1.11 (0.92, 1.35) | 0.180 |
| **Prostate cancer patients** | | | | | | |
|  | *All-cause mortality* | | | *Prostate cancer-specific mortality* | | |
|  | Q1 (1.14) | Q4 (8.52) | *P*_trend_ | Q1 (1.14) | Q4 (8.52) | *P*_trend_ |
| Excluded patients whose prostate cancer was not the first diagnosed cancer (n=159) | 1.00 (reference) | 1.24 (1.04, 1.47) | 0.004 | 1.00 (reference) | 1.33 (0.85, 2.08) | 0.100 |
| Excluded patients whose colorectal cancer was diagnosed ≤2 years after DHQ completion (n=972) | 1.00 (reference) | 1.20 (0.98, 1.45) | 0.012 | 1.00 (reference) | 1.10 (0.64, 1.87) | 0.513 |
| Excluded patients who died within 30 days after prostate cancer diagnosis (n=5) | 1.00 (reference) | 1.18 (1.00, 1.39) | 0.018 | 1.00 (reference) | 1.29 (0.83, 2.00) | 0.121 |
| Excluded patients who died within 90 days after prostate cancer diagnosis (n=18) | 1.00 (reference) | 1.18 (1.00, 1.39) | 0.019 | 1.00 (reference) | 1.32 (0.85, 2.05) | 0.098 |
| Excluded patients with extreme ultra-processed food consumption (n=267) ^b^ | 1.00 (reference) | 1.19 (1.00, 1.42) | 0.017 | 1.00 (reference) | 1.20 (0.76, 1.91) | 0.223 |
| Excluded patients with extreme energy intake (n=214) ^c^ | 1.00 (reference) | 1.19 (1.01, 1.42) | 0.013 | 1.00 (reference) | 1.23 (0.79, 1.93) | 0.177 |
| Additional adjustment on model 2 ^d^ | | | | | | |
| Intakes of fruit, vegetable, coffee, dairy, fish, whole grain, and red and processed meat | 1.00 (reference) | 1.18 (0.99, 1.41) | 0.026 | 1.00 (reference) | 1.25 (0.79, 1.99) | 0.186 |
| Intakes of dietary fiber, added sugar, saturated fatty acids, and polyunsaturated fatty acids | 1.00 (reference) | 1.16 (0.98, 1.39) | 0.033 | 1.00 (reference) | 1.37 (0.87, 2.17) | 0.074 |
| Healthy Eating Index-2015 | 1.00 (reference) | 1.15 (0.96, 1.32) | 0.061 | 1.00 (reference) | 1.13 (0.72, 1.80) | 0.352 |
| Western diet score | 1.00 (reference) | 1.19 (1.01, 1.40) | 0.015 | 1.00 (reference) | 1.28 (0.82, 1.99) | 0.128 |
| Glycemic index | 1.00 (reference) | 1.23 (1.03, 1.47) | 0.006 | 1.00 (reference) | 1.36 (0.85, 2.18) | 0.088 |
| Glycemic load | 1.00 (reference) | 1.18 (1.00, 1.39) | 0.017 | 1.00 (reference) | 1.31 (0.89, 2.08) | 0.104 |
| **Breast cancer patients** | | | | | | |
|  | *All-cause mortality* | | | *Breast cancer-specific mortality* | | |
|  | Q1 (0.63) | Q4 (6.72) | *P*_trend_ | Q1 (0.63) | Q4 (6.72) | *P*_trend_ |
| Excluded patients whose breast cancer was not the first diagnosed cancer (n=46) | 1.00 (reference) | 1.04 (0.82, 1.31) | 0.512 | 1.00 (reference) | 0.74 (0.47, 1.16) | 0.284 |
| Excluded patients whose colorectal cancer was diagnosed ≤2 years after DHQ completion (n=591) | 1.00 (reference) | 0.91 (0.70, 1.19) | 0.979 | 1.00 (reference) | 0.73 (0.44, 1.20) | 0.117 |
| Excluded patients who died within 30 days after breast cancer diagnosis (n=1) | 1.00 (reference) | 1.03 (0.82, 1.30) | 0.572 | 1.00 (reference) | 0.71 (0.45, 1.10) | 0.208 |
| Excluded patients who died within 90 days after breast cancer diagnosis (n=5) | 1.00 (reference) | 1.04 (0.83, 1.31) | 0.524 | 1.00 (reference) | 0.72 (0.46, 1.12) | 0.242 |
| Excluded patients with extreme ultra-processed food consumption (n=122) ^b^ | 1.00 (reference) | 1.06 (0.83, 1.35) | 0.427 | 1.00 (reference) | 0.75 (0.47, 1.20) | 0.339 |
| Excluded patients with extreme energy intake (n=10) ^c^ | 1.00 (reference) | 1.01 (0.81, 1.28) | 0.656 | 1.00 (reference) | 0.71 (0.45, 1.10) | 0.219 |
| Additional adjustment on model 2 ^d^ | | | | | | |
| Intakes of fruit, vegetable, coffee, dairy, fish, whole grain, and red and processed meat | 1.00 (reference) | 1.06 (0.83, 1.37) | 0.444 | 1.00 (reference) | 0.73 (0.44, 1.20) | 0.336 |
| Intakes of dietary fiber, added sugar, saturated fatty acids, and polyunsaturated fatty acids | 1.00 (reference) | 1.02 (0.80, 1.30) | 0.625 | 1.00 (reference) | 0.74 (0.46, 1.18) | 0.321 |
| Healthy Eating Index-2015 | 1.00 (reference) | 0.97 (0.76, 1.23) | 0.967 | 1.00 (reference) | 0.80 (0.50, 1.29) | 0.517 |
| Western diet score | 1.00 (reference) | 1.05 (0.83, 1.33) | 0.472 | 1.00 (reference) | 0.71 (0.45, 1.12) | 0.233 |
| Glycemic index | 1.00 (reference) | 1.06 (0.83, 1.36) | 0.438 | 1.00 (reference) | 0.78 (0.48, 1.27) | 0.454 |
| Glycemic load | 1.00 (reference) | 1.00 (0.79, 1.26) | 0.760 | 1.00 (reference) | 0.66 (0.42, 1.03) | 0.128 |

Abbreviation: quartile, Q.

^a^ Data were expressed as hazard ratios (95% confidence interval). Hazard ratios were adjusted for the following variables unless otherwise specified: age at diagnosis (years), sex (male, female; only for colorectal and lung cancers), racial/ethnic group (non-Hispanic White, non-Hispanic Black, Hispanic, others), trial arm (screening, control), body mass index (kg/m^2^), physical activity (min/week), alcohol consumption (g/day), smoking status [current (>20 cigarettes/day, 10-20 cigarettes/day, <10 cigarettes/day), former (stop smoking >15 years, stop smoking ≤15 years), never], aspirin use (yes, no), energy intake from diet (kcal/day), family history of indicated cancer (yes, no), history of diabetes (yes, no), history of hypertension (yes, no; only for all-cause mortality), and clinical covariates. For colorectal cancer, clinical covariates were cancer stage (9 categories), surgical resection (yes, no), chemotherapy (yes, no), and radiotherapy (yes, no); for lung cancer, clinical covariates were cancer stage (11 categories), surgical resection (yes, no), chemotherapy (yes, no), and radiotherapy (yes, no); for prostate cancer, clinical covariates were cancer stage (6 categories), Gleason score (2 to 10 points), PSA level closest to diagnosis (ng/mL), surgical resection (yes, no), radiotherapy (yes, no), cryosurgery or hyperthermia therapy (yes, no), and hormonal therapy (yes, no); for breast cancer, clinical covariates were cancer stage (10 categories), estrogen receptor status (positive, negative, unknown), progesterone receptor status (positive, negative, unknown), HER2 status (0, 1+, 2+, 3+, unknown), and hormone replacement therapy (current use, former use, never use, unknown).

^b^ Extreme consumption referred to top 2.5% or bottom 2.5% of ultra-processed food consumption.

^c^ Extreme energy intake referred to <800 or >4000 kcal/day for male and <500 or >3500 kcal/day for female.

^d^ All variables were treated as the continuous variable in the regression model.

| **Table S11.** Explanatory analyses on the associations of energy-adjusted consumption (daily serving) of individual ultra-processed food groups before cancer diagnosis with all-cause and cancer-specific mortality in patients with colorectal, lung, prostate, or breast cancer ^a^ | | | | | |
| --- | --- | --- | --- | --- | --- |
| Patient group | Hazard ratio (95% confidence interval) | | | | |
|  | Quartile 1 | Quartile 2 | Quartile 3 | Quartile 4 | *P*_trend_ |
| **Colorectal cancer patients** | | | | | |
| *All-cause mortality* | | | | | |
| Cereals | 1.00 (reference) | 0.81 (0.64, 1.03) | 0.90 (0.72, 1.13) | 0.82 (0.64, 1.04) | 0.689 |
| Soft drinks | 1.00 (reference) | 0.97 (0.77, 1.22) | 0.94 (0.75, 1.18) | 0.84 (0.66, 1.06) | 0.233 |
| Sauces and dressings | 1.00 (reference) | 1.22 (0.97, 1.54) | 1.07 (0.84, 1.36) | 1.05 (0.82, 1.34) | 0.700 |
| Meat and meat products | 1.00 (reference) | 0.83 (0.66, 1.05) | 0.85 (0.67, 1.08) | 0.95 (0.74, 1.21) | 0.053 |
| Salty snacks | 1.00 (reference) | 0.95 (0.75, 1.21) | 0.85 (0.67, 1.09) | 1.06 (0.83, 1.35) | 0.064 |
| Ultra-processed dairy products | 1.00 (reference) | 1.09 (0.85, 1.39) | 1.05 (0.83, 1.34) | 1.10 (0.90, 1.38) | 0.347 |
| Margarine | 1.00 (reference) | 1.13 (0.89, 1.45) | 0.97 (0.76, 1.24) | 0.86 (0.68, 1.10) | 0.305 |
| Sugary products | 1.00 (reference) | 0.95 (0.73, 1.22) | 1.12 (0.88, 1.44) | 1.10 (0.86, 1.41) | 0.894 |
| Ultra-processed fruits and vegetables | 1.00 (reference) | 0.92 (0.72, 1.18) | 0.94 (0.74, 1.19) | 1.16 (0.91, 1.49) | 0.098 |
| *Colorectal cancer-specific mortality* | | | | | |
| Cereals | 1.00 (reference) | 1.00 (0.71, 1.39) | 1.13 (0.82, 1.56) | 0.91 (0.62, 1.32) | 0.689 |
| Soft drinks | 1.00 (reference) | 1.07 (0.77, 1.50) | 1.00 (0.71, 1.40) | 0.87 (0.61, 1.23) | 0.233 |
| Sauces and dressings | 1.00 (reference) | 1.15 (0.83, 1.61) | 1.17 (0.83, 1.65) | 0.98 (0.68, 1.40) | 0.700 |
| Meat and meat products | 1.00 (reference) | 0.82 (0.58, 1.15) | 0.95 (0.66, 1.35) | 1.25 (0.88, 1.79) | 0.053 |
| Salty snacks | 1.00 (reference) | 0.91 (0.65, 1.27) | 1.02 (0.72, 1.44) | 1.31 (0.92, 1.87) | 0.064 |
| Ultra-processed dairy products | 1.00 (reference) | 1.52 (1.07, 2.15) | 1.31 (0.92, 1.86) | 1.36 (0.95, 1.95) | 0.347 |
| Margarine | 1.00 (reference) | 1.41 (0.97, 2.04) | 1.36 (0.94, 1.96) | 1.01 (0.69, 1.46) | 0.305 |
| Sugary products | 1.00 (reference) | 1.11 (0.78, 1.60) | 1.14 (0.79, 1.64) | 1.04 (0.72, 1.50) | 0.894 |
| Ultra-processed fruits and vegetables | 1.00 (reference) | 1.05 (0.73, 1.49) | 0.99 (0.70, 1.41) | 1.30 (0.92, 1.85) | 0.098 |
| **Lung cancer patients** | | | | | |
| *All-cause mortality* | | | | | |
| Cereals | 1.00 (reference) | 1.03 (0.89, 1.21) | 1.05 (0.90, 1.22) | 1.12 (0.96, 1.30) | 0.149 |
| Soft drinks | 1.00 (reference) | 0.98 (0.85, 1.14) | 0.85 (0.73, 0.99) | 1.09 (0.94, 1.26) | 0.104 |
| Sauces and dressings | 1.00 (reference) | 1.08 (0.93, 1.27) | 1.14 (0.98, 1.34) | 1.17 (1.01, 1.36) | 0.046 |
| Meat and meat products | 1.00 (reference) | 0.91 (0.78, 1.06) | 1.05 (0.90, 1.22) | 0.95 (0.81, 1.10) | 0.671 |
| Salty snacks | 1.00 (reference) | 0.97 (0.84, 1.13) | 0.98 (0.84, 1.14) | 0.94 (0.81, 1.09) | 0.427 |
| Ultra-processed dairy products | 1.00 (reference) | 0.91 (0.79, 1.06) | 0.88 (0.76, 1.03) | 0.97 (0.84, 1.13) | 0.938 |
| Margarine | 1.00 (reference) | 0.81 (0.69, 0.95) | 0.97 (0.83, 1.12) | 0.92 (0.79, 1.06) | 0.895 |
| Sugary products | 1.00 (reference) | 0.93 (0.79, 1.09) | 0.83 (0.71, 0.97) | 0.98 (0.85, 1.14) | 0.626 |
| Ultra-processed fruits and vegetables | 1.00 (reference) | 0.94 (0.81, 1.10) | 0.99 (0.84, 1.15) | 1.15 (0.99, 1.33) | 0.016 |
| *Lung cancer-specific mortality* | | | | | |
| Cereals | 1.00 (reference) | 1.05 (0.89, 1.24) | 1.03 (0.87, 1.21) | 1.13 (0.96, 1.34) | 0.151 |
| Soft drinks | 1.00 (reference) | 0.98 (0.83, 1.15) | 0.81 (0.68, 0.96) | 1.01 (0.86, 1.19) | 0.617 |
| Sauces and dressings | 1.00 (reference) | 1.17 (0.98, 1.39) | 1.14 (0.96, 1.36) | 1.23 (1.05, 1.44) | 0.033 |
| Meat and meat products | 1.00 (reference) | 0.95 (0.80, 1.12) | 1.09 (0.92, 1.29) | 0.94 (0.79, 1.11) | 0.506 |
| Salty snacks | 1.00 (reference) | 1.00 (0.85, 1.18) | 1.04 (0.88, 1.23) | 0.97 (0.83, 1.14) | 0.733 |
| Ultra-processed dairy products | 1.00 (reference) | 0.92 (0.78, 1.08) | 0.90 (0.76, 1.06) | 1.00 (0.85, 1.18) | 0.759 |
| Margarine | 1.00 (reference) | 0.80 (0.68, 0.95) | 0.89 (0.75, 1.05) | 0.88 (0.75, 1.03) | 0.518 |
| Sugary products | 1.00 (reference) | 0.82 (0.69, 0.97) | 0.83 (0.70, 0.99) | 0.96 (0.81, 1.12) | 0.536 |
| Ultra-processed fruits and vegetables | 1.00 (reference) | 0.93 (0.79, 1.11) | 1.01 (0.86, 1.20) | 1.10 (0.93, 1.29) | 0.113 |
| **Prostate cancer patients** | | | | | |
| *All-cause mortality* | | | | | |
| Cereals | 1.00 (reference) | 0.97 (0.83, 1.12) | 0.98 (0.85, 1.14) | 0.98 (0.85, 1.13) | 0.893 |
| Soft drinks | 1.00 (reference) | 0.94 (0.81, 1.08) | 0.98 (0.85, 1.13) | 1.11 (0.96, 1.28) | 0.042 |
| Sauces and dressings | 1.00 (reference) | 1.07 (0.93, 1.23) | 1.01 (0.87, 1.17) | 1.04 (0.91, 1.20) | 0.737 |
| Meat and meat products | 1.00 (reference) | 1.01 (0.86, 1.18) | 1.01 (0.87, 1.18) | 1.16 (1.00, 1.33) | 0.019 |
| Salty snacks | 1.00 (reference) | 0.86 (0.75, 1.00) | 0.88 (0.76, 1.01) | 0.97 (0.84, 1.11) | 0.089 |
| Ultra-processed dairy products | 1.00 (reference) | 0.88 (0.75, 1.02) | 0.91 (0.79, 1.06) | 0.88 (0.77, 1.00) | 0.132 |
| Margarine | 1.00 (reference) | 1.07 (0.92, 1.24) | 1.03 (0.89, 1.19) | 1.11 (0.97, 1.27) | 0.191 |
| Sugary products | 1.00 (reference) | 1.09 (0.94, 1.26) | 1.12 (0.97, 1.30) | 1.06 (0.92, 1.22) | 0.682 |
| Ultra-processed fruits and vegetables | 1.00 (reference) | 1.02 (0.88, 1.19) | 1.05 (0.90, 1.21) | 1.14 (1.00, 1.31) | 0.041 |
| *Prostate cancer-specific mortality* | | | | | |
| Cereals | 1.00 (reference) | 0.86 (0.58, 1.27) | 1.11 (0.76, 1.61) | 1.20 (0.83, 1.75) | 0.171 |
| Soft drinks | 1.00 (reference) | 1.33 (0.90, 1.97) | 1.30 (0.90, 1.88) | 1.33 (0.90, 1.96) | 0.359 |
| Sauces and dressings | 1.00 (reference) | 1.02 (0.71, 1.45) | 0.92 (0.64, 1.33) | 1.07 (0.74, 1.53) | 0.763 |
| Meat and meat products | 1.00 (reference) | 1.15 (0.74, 1.79) | 1.23 (0.82, 1.87) | 1.60 (1.08, 2.36) | 0.010 |
| Salty snacks | 1.00 (reference) | 1.06 (0.73, 1.53) | 0.85 (0.58, 1.24) | 0.82 (0.57, 1.18) | 0.195 |
| Ultra-processed dairy products | 1.00 (reference) | 0.83 (0.56, 1.23) | 0.90 (0.61, 1.32) | 1.11 (0.79, 1.56) | 0.330 |
| Margarine | 1.00 (reference) | 0.76 (0.52, 1.12) | 1.07 (0.75, 1.53) | 0.96 (0.67, 1.37) | 0.782 |
| Sugary products | 1.00 (reference) | 1.08 (0.75, 1.56) | 0.74 (0.49, 1.11) | 1.41 (1.00, 2.00) | 0.036 |
| Ultra-processed fruits and vegetables | 1.00 (reference) | 0.93 (0.62, 1.40) | 0.98 (0.67, 1.41) | 1.01 (0.71, 1.44) | 0.867 |
| **Breast cancer patients** | | | | | |
| *All-cause mortality* | | | | | |
| Cereals | 1.00 (reference) | 0.92 (0.76, 1.12) | 0.87 (0.71, 1.08) | 0.98 (0.77, 1.23) | 0.845 |
| Soft drinks | 1.00 (reference) | 1.05 (0.85, 1.29) | 1.06 (0.85, 1.32) | 0.98 (0.78, 1.23) | 0.675 |
| Sauces and dressings | 1.00 (reference) | 1.15 (0.89, 1.48) | 1.04 (0.81, 1.34) | 0.94 (0.73, 1.21) | 0.154 |
| Meat and meat products | 1.00 (reference) | 1.13 (0.93, 1.38) | 1.04 (0.84, 1.29) | 1.18 (0.94, 1.49) | 0.249 |
| Salty snacks | 1.00 (reference) | 1.26 (1.01, 1.57) | 1.04 (0.83, 1.30) | 0.92 (0.73, 1.17) | 0.091 |
| Ultra-processed dairy products | 1.00 (reference) | 0.93 (0.74, 1.19) | 1.17 (0.93, 1.48) | 1.03 (0.81, 1.30) | 0.635 |
| Margarine | 1.00 (reference) | 1.20 (0.95, 1.52) | 1.04 (0.83, 1.31) | 1.14 (0.92, 1.43) | 0.557 |
| Sugary products | 1.00 (reference) | 0.78 (0.61, 1.01) | 0.79 (0.61, 1.02) | 1.07 (0.85, 1.36) | 0.181 |
| Ultra-processed fruits and vegetables | 1.00 (reference) | 1.02 (0.82, 1.27) | 1.11 (0.88, 1.39) | 0.96 (0.77, 1.21) | 0.704 |
| *Breast cancer-specific mortality* | | | | | |
| Cereals | 1.00 (reference) | 0.87 (0.59, 1.28) | 0.73 (0.47, 1.12) | 0.72 (0.45, 1.16) | 0.156 |
| Soft drinks | 1.00 (reference) | 1.31 (0.85, 2.03) | 1.07 (0.67, 1.71) | 1.04 (0.65, 1.66) | 0.598 |
| Sauces and dressings | 1.00 (reference) | 1.62 (0.97, 2.71) | 1.27 (0.75, 2.13) | 1.20 (0.71, 2.02) | 0.708 |
| Meat and meat products | 1.00 (reference) | 1.12 (0.74, 1.67) | 1.17 (0.76, 1.78) | 1.35 (0.87, 2.11) | 0.184 |
| Salty snacks | 1.00 (reference) | 1.53 (0.98, 2.39) | 1.05 (0.67, 1.63) | 0.91 (0.57, 1.47) | 0.218 |
| Ultra-processed dairy products | 1.00 (reference) | 1.02 (0.74, 1.40) | 1.10 (0.71, 1.72) | 1.23 (0.76, 1.97) | 0.549 |
| Margarine | 1.00 (reference) | 1.36 (0.84, 2.18) | 1.12 (0.70, 1.79) | 1.04 (0.66, 1.65) | 0.599 |
| Sugary products | 1.00 (reference) | 0.74 (0.45, 1.23) | 0.83 (0.51, 1.37) | 0.78 (0.49, 1.24) | 0.571 |
| Ultra-processed fruits and vegetables | 1.00 (reference) | 0.98 (0.62, 1.54) | 1.21 (0.76, 1.93) | 1.10 (0.70, 1.72) | 0.600 |

^a^ Hazard ratios were adjusted for the following variables unless otherwise specified: age at diagnosis (years), sex (male, female; only for colorectal and lung cancers), racial/ethnic group (non-Hispanic White, non-Hispanic Black, Hispanic, others), trial arm (screening, control), body mass index (kg/m^2^), physical activity (min/week), alcohol consumption (g/day), smoking status [current (>20 cigarettes/day, 10-20 cigarettes/day, <10 cigarettes/day), former (stop smoking >15 years, stop smoking ≤15 years), never], aspirin use (yes, no), energy intake from diet (kcal/day), family history of indicated cancer (yes, no), history of diabetes (yes, no), history of hypertension (yes, no; only for all-cause mortality), and clinical covariates. For colorectal cancer, clinical covariates were cancer stage (9 categories), surgical resection (yes, no), chemotherapy (yes, no), and radiotherapy (yes, no); for lung cancer, clinical covariates were cancer stage (11 categories), surgical resection (yes, no), chemotherapy (yes, no), and radiotherapy (yes, no); for prostate cancer, clinical covariates were cancer stage (6 categories), Gleason score (2 to 10 points), PSA level closest to diagnosis (ng/mL), surgical resection (yes, no), radiotherapy (yes, no), cryosurgery or hyperthermia therapy (yes, no), and hormonal therapy (yes, no); for breast cancer, clinical covariates were cancer stage (10 categories), estrogen receptor status (positive, negative, unknown), progesterone receptor status (positive, negative, unknown), HER2 status (0, 1+, 2+, 3+, unknown), and hormone replacement therapy (current use, former use, never use, unknown). All ultra-processed food groups were mutually adjusted, with the consumption of each food group covariate treated as the continuous variable in regression models.
